# Induction of Interferon‐γ and Tissue Inflammation by Overexpression of Eosinophil Cationic Protein in T Cells and Exosomes

**DOI:** 10.1002/art.41920

**Published:** 2021-12-09

**Authors:** Huai‐Chia Chuang, Ming‐Han Chen, Yi‐Ming Chen, Yi‐Ru Ciou, Chia‐Hsin Hsueh, Ching‐Yi Tsai, Tse‐Hua Tan

**Affiliations:** ^1^ National Health Research Institutes Zhunan Taiwan; ^2^ Taipei Veterans General Hospital and National Yang Ming Chiao Tung University School of Medicine Taipei Taiwan; ^3^ Taichung Veterans General Hospital Taichung Taiwan; ^4^ National Health Research Institutes, Zhunan, Taiwan, and Baylor College of Medicine Houston Texas

## Abstract

**Objective:**

T cells play a critical role in the pathogenesis of systemic lupus erythematosus (SLE). Serum‐derived exosomes are increased in SLE patients and are correlated with disease severity. This study was undertaken to investigate whether T cell–derived exosomal proteins play a role in SLE pathogenesis.

**Methods:**

We characterized proteins in T cell–derived exosomes from SLE patients and healthy controls by MACSPlex exosome analysis and proteomics. To study the potential pathogenic functions of the exosomal protein identified, we generated and characterized T cell–specific transgenic mice that overexpressed that protein in T cells.

**Results:**

We identified eosinophil cationic protein (ECP, also called human RNase III) as overexpressed in SLE T cell–derived exosomes. T cell–specific ECP–transgenic mice (n = 5 per group) displayed early induction of serum interferon‐γ (IFNγ) levels (*P* = 0.062) and inflammation of multiple tissue types. Older T cell–specific ECP–transgenic mice (n = 3 per group) also displayed an increase in follicular helper T cell and plasma B cell numbers, and in autoantibody levels (*P* < 0.01). Single‐cell RNA sequencing showed the induction of IFNγ messenger RNA (*P* = 2.2 × 10^‐13^) and inflammatory pathways in ECP‐transgenic mouse T cells. Notably, adoptively transferred ECP‐containing exosomes stimulated serum autoantibody levels (*P* < 0.01) and tissue IFNγ levels in the recipient mice (n = 3 per group). The transferred exosomes infiltrated into multiple tissues of the recipient mice, resulting in hepatitis, nephritis, and arthritis.

**Conclusion:**

Our findings indicate that ECP overexpression in T cells or T cell–derived exosomes may be a biomarker and pathogenic factor for nephritis, hepatitis, and arthritis associated with SLE.

## INTRODUCTION

Autoimmune diseases are chronic, debilitating, incurable, and life‐threatening diseases; patients with autoimmune diseases need to receive treatments throughout their life. Patients with systemic lupus erythematosus (SLE) may have inflammation and tissue damage in the liver, kidney, skin, lung, joint, central nervous system, and other organs (1). Despite recent advances in biologic therapies (such as tocilizumab/anti–interleukin‐6 receptor [anti–IL‐6R] antibody and adalimumab/anti–tumor necrosis factor [anti‐TNF] antibody), 30% of patients with rheumatoid arthritis (RA) and 26–38% of patients with ankylosing spondylitis fail to respond to all therapies (2, 3, 4). Furthermore, the majority of patients who show improvement after treatment do not achieve complete remission, and their response to therapy may diminish over time (5, 6). Diagnosis and treatment of SLE are challenging due to complex symptoms and a lack of effective therapeutics (1, 6). Identification of novel therapeutic targets will help future development of effective treatments for SLE. Moreover, novel diagnostic/prognostic biomarkers will help to stratify patients who are likely to respond to a specific drug, leading to precision medicine.

T cells promote autoimmune diseases by inducing autoantibody production and inflammatory responses (7, 8, 9, 10, 11, 12). Effector memory T cell and Th17 cell numbers are increased in SLE patients (7, 13, 14). The Th1:Th2 cell ratio is also enhanced in SLE patients (15, 16). Th1‐secreted interferon‐γ (IFNγ) and TNF contribute to macrophage activation and damage of multiple tissue types (15). Th17‐secreted IL‐17A is a key pathogenic cytokine in inflammation and autoimmune responses (17, 18). Th17 cells recruit macrophages and dendritic cells to inflammation sites; Th17 cells also facilitate B cell activation and autoantibody production (17). Conversely, the Treg cell population is decreased in SLE patients (19). Thus, T cell hyperactivation plays a critical role in the pathogenesis of SLE.

Cell‐derived exosomes directionally deliver proteins, amino acids, microRNA, or metabolites to targeted cells or tissues to modulate cell or tissue characteristics (20, 21, 22, 23). Moreover, T cell–derived exosomal microRNAs modulate immune responses (22, 24, 25). The number of exosomes in the sera of SLE patients is correlated with disease severity (26). These serum‐derived exosomes from SLE patients induce the production of proinflammatory cytokines by impacted peripheral blood mononuclear cells from healthy individuals (26). To date, the surface proteins and intra‐exosomal proteins of exosomes in SLE patients, as well as the regulatory mechanisms of exosomal protein–induced inflammation in SLE patients, remain unclear.

Eosinophil cationic protein (ECP; also called human RNase III) is a defense protein; eosinophils release ECP during degranulation against bacterial or parasitic infection (27). ECP disrupts the bacteria membrane through binding to lipopolysaccharide or other bacterial cell wall components (27). In addition to being increased during infection, ECP levels are increased in human patients with allergic asthma or atopic dermatitis (28). Moreover, ECP treatment induces mammalian cell necrosis and inhibits cell growth and proliferation (29, 30, 31). ECP treatment also induces cell apoptosis through TNF–caspase signaling (32). To date, the roles of ECP in T cell function and autoimmune disease pathogenesis remain unknown. In this study, we characterized T cell–derived exosomes from SLE patients and identified ECP as a pathogenic exosomal protein.

## MATERIALS AND METHODS

### Human subjects

This study was conducted in accordance with the Declaration of Helsinki. A total of 50 individuals, including 24 healthy controls, 24 SLE patients, and 2 RA patients, were enrolled. Nine of the SLE patients and the 2 RA patients had been referred to the Division of Immunology and Rheumatology at Taichung Veterans General Hospital in Taiwan. The remaining 15 SLE patients had been referred to the Division of Immunology and Rheumatology at Taipei Veterans General Hospital in Taiwan. Characteristics of the SLE patients are provided in Supplementary Table 1, available on the *Arthritis & Rheumatology* website at http://onlinelibrary.wiley.com/doi/10.1002/art.41920/abstract.

The collection of peripheral blood from healthy controls and patients, and the experiments, were approved by the ethics committees of Taichung Veterans General Hospital (#SE17193B) and Taipei Veterans General Hospital (2017‐06‐003BC). All study participants provided written informed consent prior to enrollment.

### Mice

All animal experiments were performed in the AAALAC‐accredited animal housing facilities at the National Health Research Institutes (NHRI). All mice were used according to protocols and guidelines approved by the Institutional Animal Care and Use Committee of NHRI. The T cell–specific human ECP–transgenic mouse line was generated at the NHRI Transgenic Mouse Core. The C57BL/6J mouse line (catalog no. 000664), MRL/MpJ mouse line (catalog no. 00486), and the autoimmune lupus model MRL/MpJ‐Fas^*lpr*^ mouse line (catalog no. 000485) were purchased from The Jackson Laboratory. The experiments in this study were performed on sex‐matched, 5–38‐week‐old littermates. For T cell development analyses, 5‐week‐old, sex‐matched mice were used. All mice used in this study were maintained in temperature‐controlled and pathogen‐free cages.

### Generation of T cell–specific ECP–transgenic (Lck‐ECP–transgenic) mice

A full‐length human ECP coding sequence and a FLAG‐tag coding sequence were placed downstream of the proximal Lck promoter, which drives gene expression specifically in T cells (33, 34, 35). The transgenic mouse line on a C57BL/6J background was generated using pronuclear microinjection at the NHRI Transgenic Mouse Core.

### Reagents and antibodies

Anti‐human ECP antibody (catalog no. E‐AB‐14971) was purchased from Elabscience; anti‐CD9 antibody (catalog no. ab92726) was purchased from Abcam. An ExoSparkler Exosome Membrane Labeling Kit (green) was purchased from Dojido Molecular Technologies. Alexa 647–conjugated donkey anti‐mouse IgG antibody was purchased from ThermoFisher. PerCP‐conjugated anti‐mouse CD3 (clone 145‐2C11), Pacific Blue–conjugated anti‐mouse CD4 (clone RM4‐5), phycoerythrin (PE)–conjugated anti‐mouse CXCR5 (clone 2G8), PerCP–Cy5.5–conjugated anti‐mouse B220 (clone RA3‐6B2), and PE‐conjugated anti‐mouse CD138 (clone 281‐2) antibodies were purchased from BD Biosciences. PE‐conjugated anti‐mouse CD44 (clone IM7), fluorescein isothiocyanate–conjugated anti‐mouse CD62L (clone MEL‐14), Pacific Blue–conjugated anti‐mouse CD21 (clone 7E9), and PE‐conjugated anti‐mouse CD23 (clone B3B4) antibodies were purchased from BioLegend. Alexa 488–conjugated anti‐mouse IL‐21 (bs‐2621R‐A488) antibody was purchased from Bioss. Enzyme‐linked immunosorbent assay (ELISA) kits for IL‐1β, IL‐6, and TNF were purchased from eBioscience. ELISA kits for antinuclear antibody (ANA), rheumatoid factor (RF), and anti–double‐stranded DNA (anti‐dsDNA) antibody were purchased from Alpha Diagnostic. The ELISA kit for human ECP was purchased from CUSABIO.

### T cell purification

Primary murine T cells were negatively selected from the spleen, lymph nodes, or peripheral blood of mice using magnetically coupled antibodies against CD11b (BD Biosciences), CD11c (BD Biosciences), B220 (BD Biosciences), CD49b (BioLegend), and Ter‐119 (BioLegend) as described previously (35). For human T cell purification, peripheral blood T cells were negatively selected from 10 ml of whole blood from participants using a cocktail of biotin‐conjugated antibodies against CD14 (eBioscience), CD11b (BioLegend), CD19 (eBioscience), and CD235a (eBioscience) on a magnetic cell separation column (Miltenyi Biotec) as described previously (14).

### Isolation of T cell–derived exosomes

Human or murine T cells (8 × 10^6^) were cultured in 2 ml RMPI 1640 medium for 96 hours without any stimulation. To remove cell debris, supernatants were subjected to centrifugation at 13,000 rpm for 15 minutes. T cell–derived exosomes were precipitated from supernatants by ExoQuick (System Biosciences). For isolation of CD9+ and C63+ exosomes, precipitated T cell exosomes were resuspended in phosphate buffered saline (PBS) and then isolated using anti‐CD9 or anti‐CD63 magnetic beads (System Biosciences). CD9+ and CD63+ exosomes were then eluted with 20 μl exosome elution buffer (System Biosciences).

### Adoptive transfer of T cell–derived exosomes

Murine T cells (8 × 10^6^) from wild‐type or Lck‐ECP–transgenic mice were cultured in 2 ml RMPI 1640 medium for 96 hours without any stimulation. To remove cell debris, supernatants were subjected to centrifugation at 13,000 rpm for 15 minutes. T cell–derived exosomes were precipitated from supernatants by ExoQuick (System Biosciences). Exosomes from 12 ml of medium were suspended in 300 μl of PBS and then intravenously injected into 3 recipient mice (100 μl/mouse) every 3 days for 9–30 days. For confocal microscopy analysis, exosomes were labeled with green fluorescent dye using an ExoSparkler Exosome Membrane Labeling Kit.

### Multiplex exosome flow cytometry assay

To characterize potential surface proteins on T cell–derived exosomes, exosomes were precipitated from T cell supernatants and subjected to bead‐based multiplex exosome flow cytometry assay using a MACSPlex Exosome Kit (human; Miltenyi Biotec). The kit contains capture beads conjugated with individual antibodies against 37 known surface markers on different exosomes. The captured exosome signals were analyzed using a BD Canto II flow cytometer.

### Single‐cell RNA‐sequencing data analysis

Murine T cells were purified from the spleens and lymph nodes of wild‐type and Lck‐ECP–transgenic mice. T cells were analyzed using a BD Rhapsody Single‐Cell Analysis System. The single‐cell RNA‐sequencing data were analyzed using BD SeqGeq software (BD Biosciences) and the R package Seurat. Dimensionality reduction was performed using Uniform Manifold Approximation and Projection; clustering analysis was performed according to individual subsets of variable genes.

### Liquid chromatography tandem mass spectrometry (LC‐MS/MS) and data analysis

For identification of proteins in T cell–derived exosomes, exosomal proteins were digested with trypsin and subjected to LC‐MS/MS analyses on an LTQ Orbitrap Elite hybrid mass spectrometer as described previously (36). The peptide data were analyzed by Mascot MS/MS Ion Search (Matrix Science) with the following conditions: peptide mass tolerance 20 parts per million; fragment MS/MS tolerance 0.6 daltons; allow up to 1 missed cleavage; peptide charge 2+, 3+, and 4+.

### Statistical analysis

In vivo experiments were conducted using distinct samples; in vitro experiments were performed at least 3 times. Statistical analysis was performed in Excel, SPSS, or BD SeqGeq. Comparisons between 2 groups were conducted using Student's unpaired 2‐tailed or 1 tailed *t*‐test and Wilcoxon's rank sum test. *P* values less than 0.05 were considered significant.

## RESULTS

### Identification of CD9 and CD63 surface markers in T cell–derived exosomes from SLE patients

**Figure 1:**
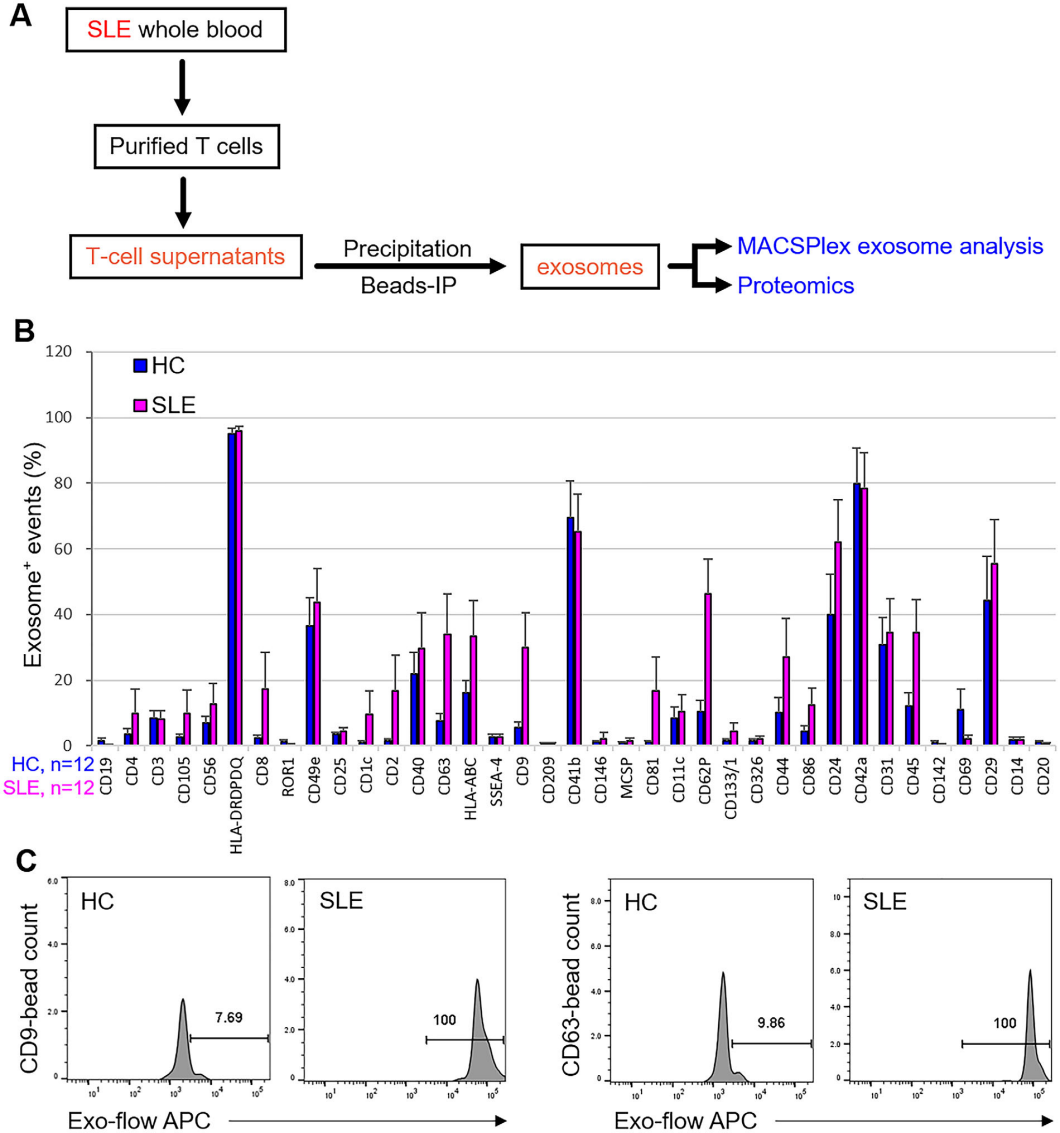
Increased numbers of T cell–derived CD9+ and CD63+ exosomes in patients with systemic lupus erythematosus (SLE). **A**, Experimental design for the characterization of SLE‐enriched T cell–derived exosomes. IP = immunoprecipitation. **B**, MACSPlex exosome analysis of T cell–derived exosomes from 12 SLE patients and 12 healthy controls (HCs). Thirty‐seven individual surface markers of exosomes were identified using a BD FACSCanto II flow cytometer. Bars show the mean ± SEM. **C**, Histograms showing the results of MACSPlex analysis of CD9+ and CD63+ exosomes from a representative healthy control and a representative SLE patient. Values are the percent allophycocyanin (APC) positive.

Peripheral blood T cells from SLE patients and healthy controls were purified and cultured for 72 hours prior to collection of T cell–derived exosomes. To identify exosomal surface proteins enriched in SLE patients, exosomes in T cell supernatants were subjected to MACSPlex assays (Figure 1A). Exosomes were captured by 37 individual surface marker antibodies conjugated with fluorescent beads, and subjected to flow cytometric analysis (Supplementary Figure 1, available on the *Arthritis & Rheumatology* website at http://onlinelibrary.wiley.com/doi/10.1002/art.41920/abstract). The numbers of T cell–derived exosomes were drastically increased in the supernatants of T cells from SLE patients (n = 12) compared to those from healthy controls (n = 12). Among 37 surface proteins, 16 exosomal surface proteins were increased in SLE patients (Figure 1B). The numbers of T cell–derived CD9+, CD63+, CD62P+, and CD45+ exosomes in SLE patients were high (with signals of >20%) and significantly increased compared to those in healthy controls (Figures 1B and C). CD9 and CD63 are 2 well‐known exosomal surface markers; interestingly, T cell–derived CD9+ and CD63+ exosomes had the highest fold induction in SLE patients compared to controls.

### Presence of ECP in T cell–derived exosomes from patients with SLE

**Figure 2:**
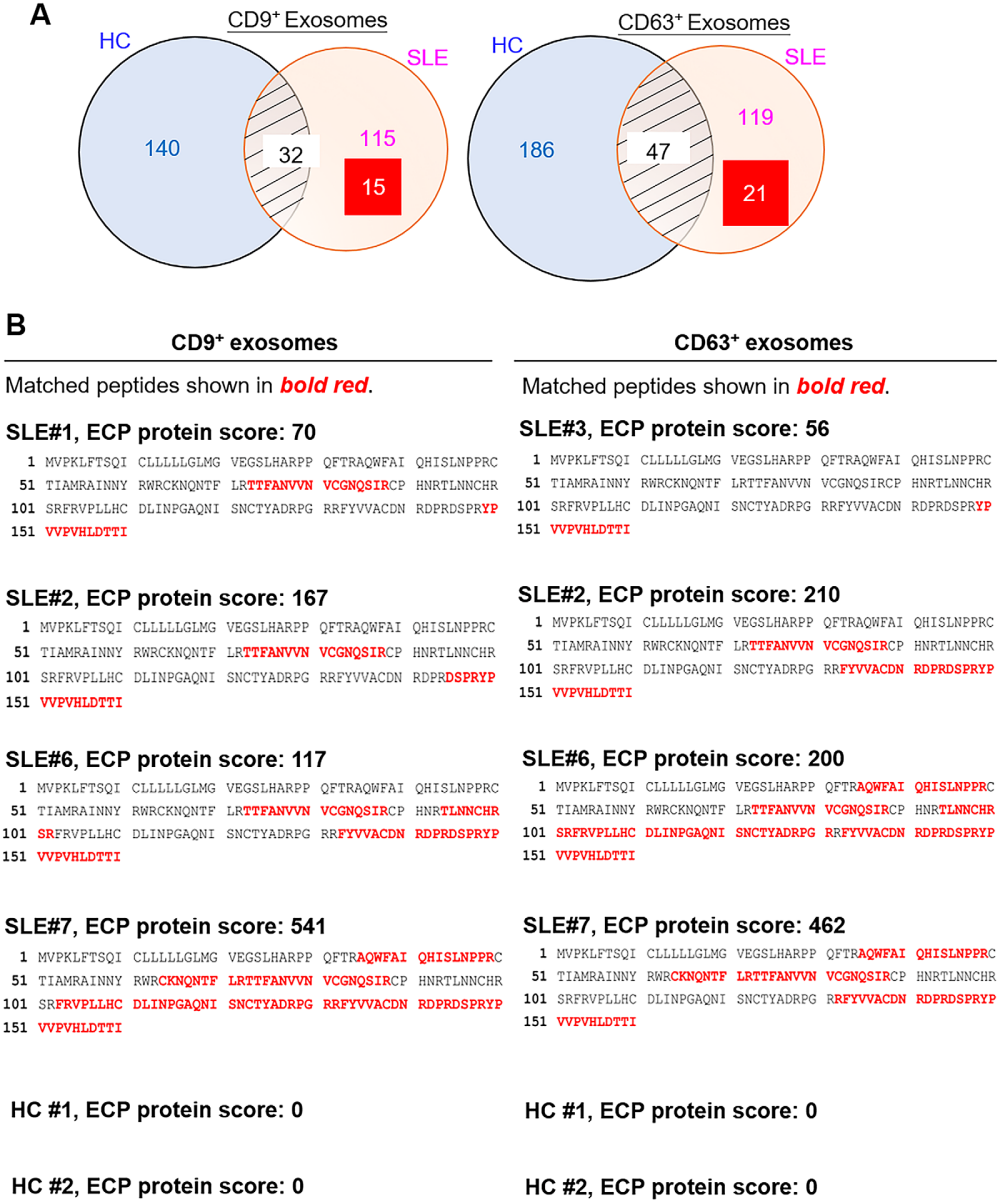
Induction of eosinophil cationic protein (ECP) in T cell–derived CD9+ and CD63+ exosomes from patients with systemic lupus erythematosus (SLE). **A**, Venn diagrams showing the numbers of proteins that were enriched in T cell–derived CD9+ or CD63+ exosomes from healthy controls (HCs) only, both healthy controls and SLE patients, and patients with SLE only. Exosomal proteins were identified by mass spectrometry–based protein sequencing. Many of the proteins identified were also enriched in exosomes from patients with rheumatoid arthritis (RA). Values in the rectangles are the number of exosomes that were enriched in SLE patients but not RA patients. **B**, Identification of ECP by mass spectrometry (MS)–based protein sequencing of T cell–derived CD9+ and CD63+ exosomes from 5 SLE patients (patients 1, 2, 3, 6, and 7). ECP was not detected in T cells from 2 healthy controls (controls 1 and 2). The protein score is the sum of the highest ion scores found by Mascot MS/MS Ion Search for each distinct peptide.

To characterize the properties of T cell–derived exosomes from SLE patients, T cell–derived CD9+ and CD63+ exosomes isolated from SLE patients and healthy controls were analyzed by mass spectrometry–based proteomics. The proteomics data showed that 130 and 140 proteins were overexpressed in T cell–derived CD9+ exosomes and CD63+ exosomes, respectively, from SLE patients but not in those from healthy controls (Figure 2A). Moreover, many of the exosomal proteins identified were also observed in RA‐enriched exosomes; only 15 CD9+ exosomal proteins and 21 CD63+ exosomal proteins were selectively enriched in SLE patients but not RA patients (Figure 2A). The identified SLE‐enriched T cell exosomal proteins encompassed protein kinases, protein phosphatases, and metabolic enzymes (Supplementary Table 2, available on the *Arthritis & Rheumatology* website at http://onlinelibrary.wiley.com/doi/10.1002/art.41920/abstract).

Notably, one exosomal protein, ECP, which had the highest protein scores, was detected in T cell exosomes from all 5 SLE patient samples included in the mass spectrometry analysis (Figure 2B and Supplementary Table 2). ECP was also determined to be enriched in SLE T cells by proteomics analysis (Supplementary Figure 2A, available on the *Arthritis & Rheumatology* website at http://onlinelibrary.wiley.com/doi/10.1002/art.41920/abstract), whereas soluble ECP levels were not increased in the sera of SLE patients (Supplementary Figure 2B). These data suggest that T cell–derived exosomal ECP may be a biomarker for SLE. Thus, the SLE T cell–enriched exosomal protein ECP was selected for further characterization.

### Development of severe inflammation in Lck‐ECP–transgenic mice

**Figure 3:**
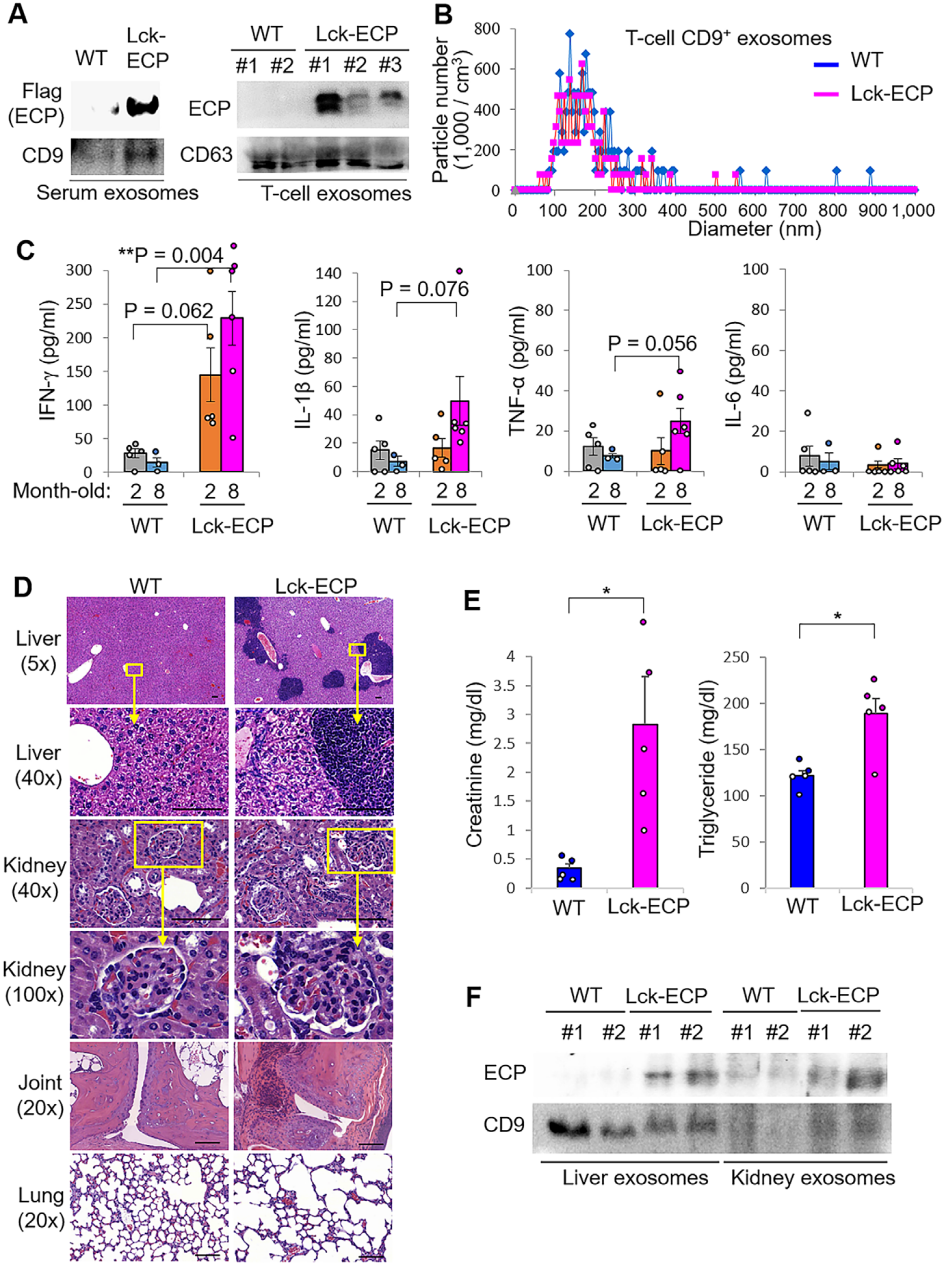
Spontaneous development of severe inflammation in T cell–specific eosinophil cationic protein (ECP)–transgenic (Lck‐ECP–transgenic) mice. **A**, Immunoblots showing FLAG‐tagged ECP and CD9 protein levels in serum exosomes and T cell–derived exosomes from 3 Lck‐ECP–transgenic mice and 2 wild‐type (WT) mice. **B**, ZetaView analysis of particle numbers and sizes of CD9+ extracellular vesicles in supernatants from WT and Lck‐ECP–transgenic mouse T cells. Extracellular vesicles were isolated by ExoQuick‐TC. **C**, Serum levels of interferon‐γ (IFNγ), interleukin‐1β (IL‐1β), tumor necrosis factor (TNF), and IL‐6 in 2‐month‐old mice (n = 5 per group) and 8‐month‐old mice (n = 3 WT mice and 6 Lck‐ECP–transgenic mice), determined by enzyme‐linked immunosorbent assay. Each symbol represents an individual mouse; bars show the mean ± SEM. **D**, Hematoxylin and eosin–stained sections of the liver, kidney, joint, and lung from 8‐month‐old Lck‐ECP–transgenic and WT mice. The bottom panels for liver and kidney show higher‐magnification views of the boxed areas in the top panels. Bars = 100 μm. **E**, Serum levels of creatinine and triglyceride in 38‐week‐old WT and Lck‐ECP–transgenic mice (n = 5 per group), measured by serum chemistry assay. Each symbol represents an individual mouse; bars show the mean ± SEM. **F**, Immunoblot showing FLAG‐tagged ECP and CD9 protein levels in exosomes isolated from the liver and kidney tissues of WT and Lck‐ECP–transgenic mice. * = *P* < 0.05; ** = *P* < 0.01, by Student's 2‐tailed *t*‐test.

To study whether SLE‐enriched exosomal ECP plays an important role in the pathogenesis of SLE, we generated and characterized Lck‐ECP–transgenic mice (Supplementary Figures 3A and B, available on the *Arthritis & Rheumatology* website at http://onlinelibrary.wiley.com/doi/10.1002/art.41920/abstract). ECP was successfully overexpressed in T cells (Supplementary Figure 3C) and T cell–derived exosomes from Lck‐ECP–transgenic mice (Figure 3A). T cell–derived exosomes were mostly <200 nm in diameter (Figure 3B and Supplementary Figure 4, available on the *Arthritis & Rheumatology* website at http://onlinelibrary.wiley.com/doi/10.1002/art.41920/abstract). Four‐week‐old Lck‐ECP–transgenic mice displayed normal T cell and B cell development in the thymus and bone marrow, respectively (Supplementary Figure 5, available on the *Arthritis & Rheumatology* website at http://onlinelibrary.wiley.com/doi/10.1002/art.41920/abstract). The peripheral CD4+ cell population was modestly increased, but the CD8+ cell population was decreased, in Lck‐ECP–transgenic mice (Supplementary Figure 5).

To study whether ECP overexpression in T cells leads to inflammatory responses in mice, we monitored serum cytokine levels in mice. Two‐month‐old Lck‐ECP–transgenic mice spontaneously developed increased serum levels of the proinflammatory cytokine IFNγ compared to wild‐type mice (Figure 3C). Serum levels of TNF, IL‐1β, and IL‐6 were not significantly increased in 2‐month‐old Lck‐ECP–transgenic mice, whereas serum levels of IL‐1β and TNF were increased in 8‐month‐old Lck‐ECP–transgenic mice (*P* = 0.076 and *P* = 0.056, respectively), compared to wild‐type mice of the same age (Figure 3C). Moreover, 10‐month‐old Lck‐ECP–transgenic mice showed severe inflammation of the liver, kidney, and joint, detected by histologic staining (Figure 3D). Increased serum levels of creatinine and triglyceride also suggest the development of nephritis and hepatitis, respectively, in Lck‐ECP–transgenic mice (Figure 3E). To study whether ECP‐containing exosomes infiltrate into the inflamed tissues of the liver and kidney, exosomes were isolated from mouse tissue samples and subjected to immunoblotting. The results verified that Lck‐ECP–transgenic mouse tissues contained ECP‐positive exosomes (Figure 3F). These results suggest that Lck‐ECP–transgenic mice spontaneously develop inflammation of multiple tissue types.

### Induction of inflammatory responses in recipient mice after adoptive transfer of T cell–derived exosomes from Lck‐ECP–transgenic mice

**Figure 4:**
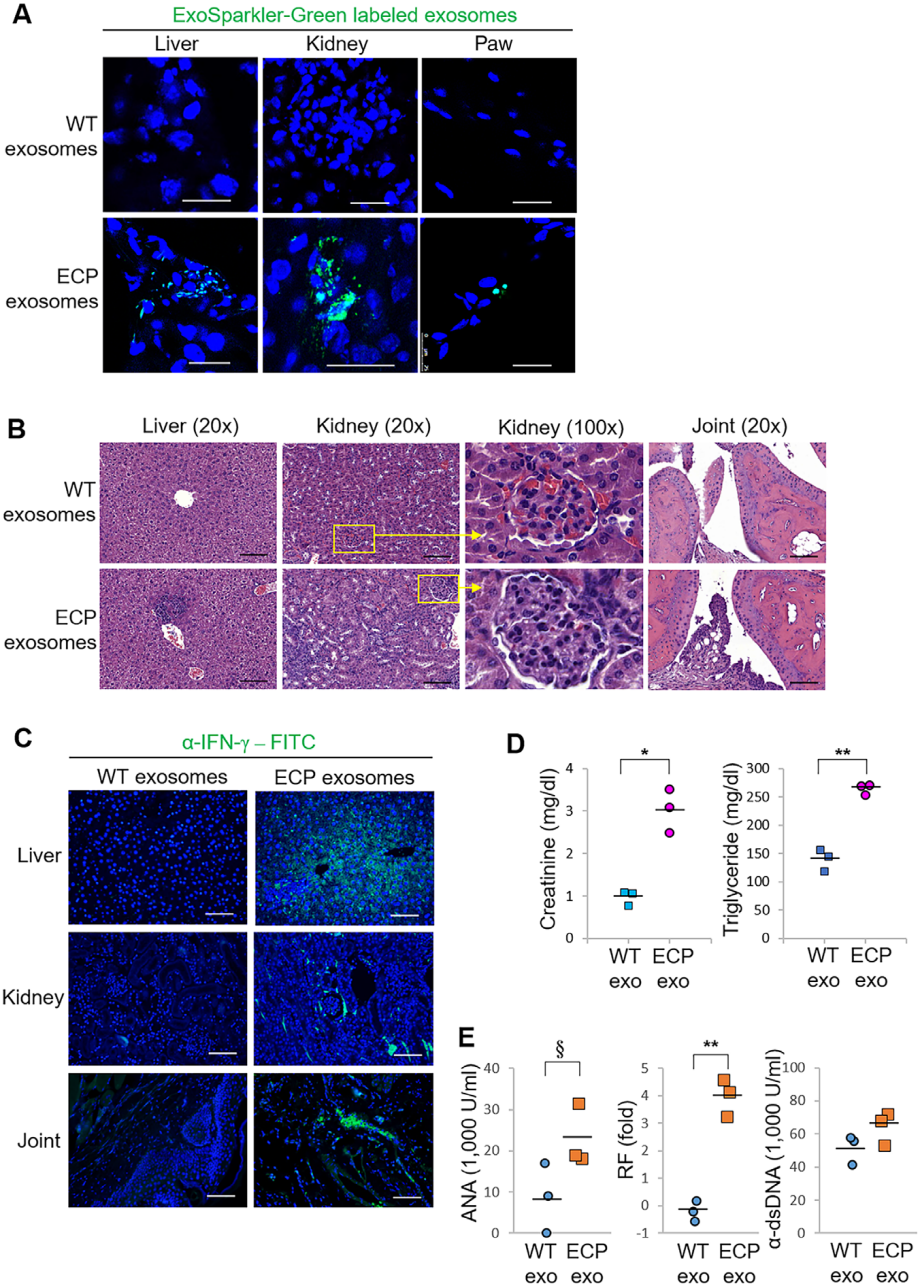
Inflammatory responses in WT mice after adoptive transfer of T cell–derived exosomes (exo) from Lck‐ECP–transgenic mice. **A**, Confocal microscopy analysis of fluorescent dye–labeled exosomes (green) in frozen sections of the liver, kidney, and paw from a WT mouse and an ECP exosome recipient mouse. Exosomes were isolated from the supernatants of WT or Lck‐ECP–transgenic mouse T cells by ExoQuick‐TC. Labeled exosomes were adoptively transferred into recipient mice by intravenous (IV) injection every 3 days for 9 days. Cell nuclei were stained with DAPI. Bars = 25 μm; original magnification × 630. **B**–**E**, Exosomes derived from WT or Lck‐ECP–transgenic mouse T cells (n = 3 per group) were adoptively transferred into recipient mice by IV injection every 3 days for 30 days. **B**, Hematoxylin and eosin–stained sections of the liver, kidney, and joint from recipient mice. Bars = 100 μm. Right panels for kidney sections show a higher‐magnification view of the boxed areas in the left panels. **C**, Immunohistochemical staining of fluorescein isothiocyanate (FITC)–conjugated anti–interferon‐γ (anti‐IFNγ) antibody (green) in paraffin‐embedded sections of the liver, kidney, and joint from recipient mice. Cell nuclei were stained with DAPI. Bars = 100 μm. **D**, Serum creatinine and triglyceride levels in recipient mice, determined by serum chemistry assays. **E**, Serum antinuclear antibody (ANA), rheumatoid factor (RF), and anti–double‐stranded DNA (anti‐dsDNA) antibody levels in recipient mice, determined by enzyme‐linked immunosorbent assay. In **D** and **E**, each symbol represents an individual mouse; bars show the mean. * = *P* < 0.05; ** = *P* < 0.01, by Student's 2‐tailed *t*‐test; § = *P* < 0.05 by Student's 1‐tailed *t*‐test. See Figure 3 for other definitions.

To demonstrate the pathologic functions of ECP‐containing exosomes, exosomes from purified Lck‐ECP–transgenic mouse T cells were adoptively transferred into wild‐type recipient mice through intravenous injection. Confocal microscopy showed that the exosomes derived from T cells from Lck‐ECP–transgenic mice (referred to hereafter as ECP exosomes) infiltrated into the tissues of the liver, kidney, and paw of the wild‐type recipient mice (Figure 4A). After 4 weeks of adoptive transfer of ECP exosomes, recipient mice displayed induction of infiltrating immune cells in the liver, kidney, and joint synovium (Figure 4B). Hematoxylin and eosin staining suggested the development of hepatitis, nephritis, and arthritis in ECP exosome–recipient mice (Figure 4B).

Consistent with the early induction of serum IFNγ levels in Lck‐ECP–transgenic mice, ECP exosomes also stimulated serum IFNγ levels in wild‐type recipient mice (Supplementary Figure 6, available on the *Arthritis & Rheumatology* website at http://onlinelibrary.wiley.com/doi/10.1002/art.41920/abstract). Liver, kidney, and joint tissues from ECP exosome–recipient mice also showed high IFNγ levels (Figure 4C). Moreover, adoptive transfer of ECP exosomes stimulated serum creatinine and triglyceride levels in wild‐type recipient mice (Figure 4D), suggesting the development of nephritis and hepatitis, respectively, in the recipient mice. Furthermore, adoptive transfer of ECP exosomes induced IgG autoantibody in tissues from the wild‐type recipient mice, suggesting immune complex deposition (Supplementary Figure 7, available on the *Arthritis & Rheumatology* website at http://onlinelibrary.wiley.com/doi/10.1002/art.41920/abstract). Serum levels of ANA and RF, but not anti‐dsDNA, were also increased in the recipient mice (Figure 4E), suggesting an induction of autoimmune response by ECP exosomes.

Next, we examined whether MRL/MpJ‐Fas^*lpr*^ mice in the autoimmune lupus model also harbor ECP‐containing exosomes. Consistent with the results described above, we found that ECP levels were indeed induced in serum exosomes and T cell–derived exosomes from MRL/MpJ‐Fas^*lpr*^ mice compared to those of wild‐type mice (Supplementary Figure 8, available on the *Arthritis & Rheumatology* website at http://onlinelibrary.wiley.com/doi/10.1002/art.41920/abstract). Taken together, these results suggest that T cell–derived exosomal ECP contributes to inflammatory responses.

### Induction of growth hormone (GH), TNF superfamily (TNFSF8), and Titin by overexpression of ECP in T cells

**Figure 5:**
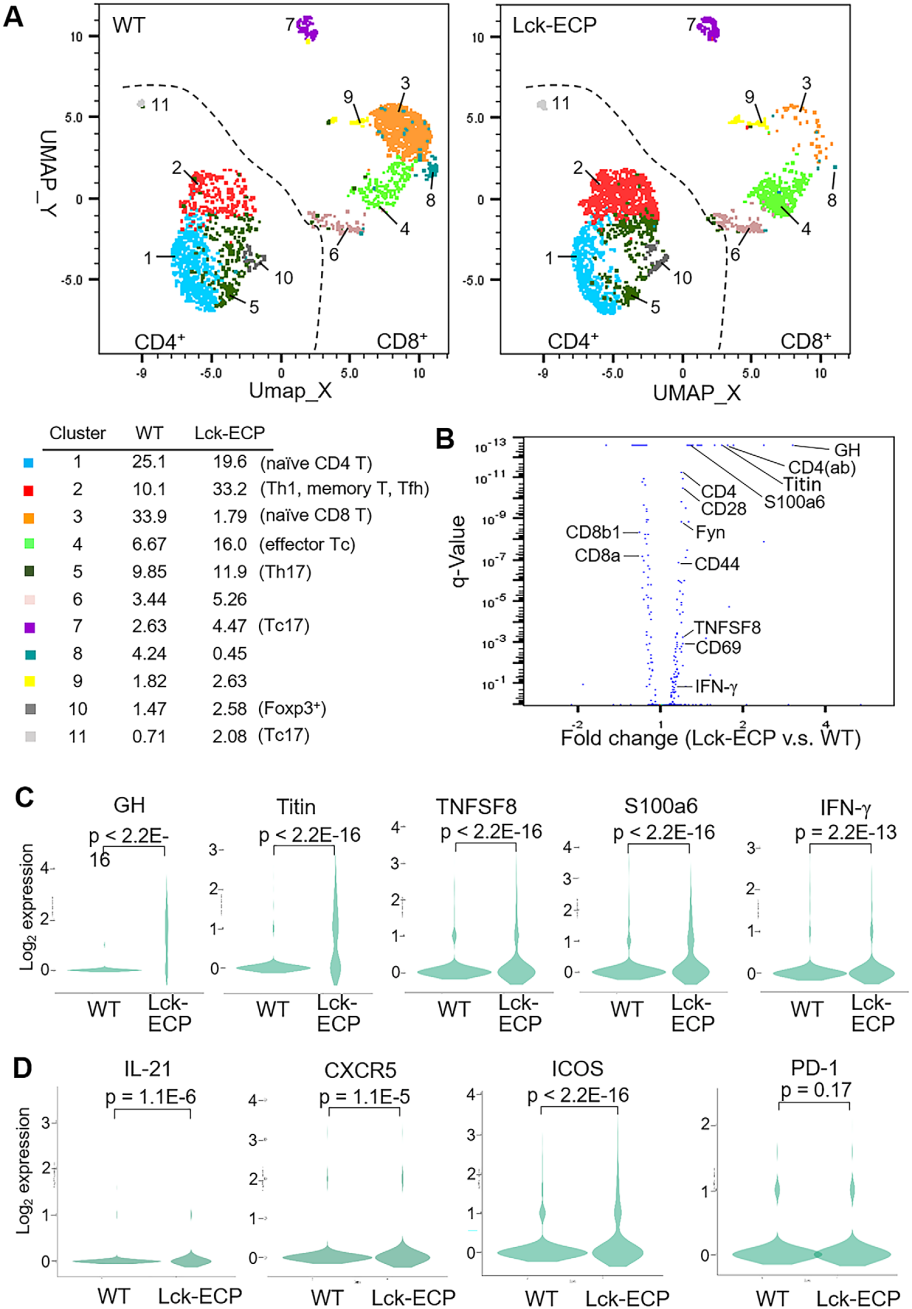
Induction of tumor necrosis factor (TNF) signaling and interferon‐γ (IFNγ) production by overexpression of ECP in T cells. **A**, Distribution and classification of T cells from Lck‐ECP–transgenic and WT mice. Data were visualized using Uniform Manifold Approximation and Projection (UMAP). Values listed for each cluster are the percentage of the cell subset among all T cells. **B**, Volcano plot showing the selected differentially expressed genes (DEGs) in Lck‐ECP–transgenic versus WT mouse T cells. The q values were determined by Fisher's exact test. **C**, Violin plots showing the expression of selected DEGs in WT and Lck‐ECP–transgenic mouse T cells. *P* values were determined by Wilcoxon's rank sum test. **D**, Violin plots showing the expression of follicular helper T (Tfh) cell markers (interleukin‐21 [IL‐21], CXCR5, inducible costimulator [ICOS], and programmed death 1 [PD‐1]) in WT and Lck‐ECP–transgenic mouse T cells. *P* values were determined by Wilcoxon's rank sum test. ab = antibody; GH = growth hormone; TNFSF8 = tumor necrosis factor superfamily 8 (see Figure 3 for other definitions).

To understand the underlying T cell responses that drive spontaneous inflammation in Lck‐ECP–transgenic mice, T cells from 8‐month‐old Lck‐ECP–transgenic mice were isolated and subjected to single‐cell RNA sequencing. Dimensionality reduction/clustering analyses by Seurat grouped T cells from Lck‐ECP and wild‐type mice into 11 distinct clusters according to gene expression (Figure 5A). Clusters 1, 2, 5, 10, and 11 were identified as CD4+ T cells, while clusters 3, 4, 6, 7, 8, and 9 were identified as CD8+ T cells (Figure 5A). According to the expression levels of CD44 and CD62L (Supplementary Figure 9A, available on the *Arthritis & Rheumatology* website at http://onlinelibrary.wiley.com/doi/10.1002/art.41920/abstract), cluster 2 was mainly memory T cells, clusters 1 and 3 were identified as naive T cells, and cluster 4 was identified as effector T cells.

ECP overexpression in T cells resulted in decreased numbers of naive CD8+ T cells (cluster 3), increased numbers of effector CD8+ T cells (cluster 4), and increased numbers of memory CD4+ T cells (cluster 2) (Figure 5A). Consistent with these findings, flow cytometry data showed an increase in the numbers of effector memory T cells (CD62L−CD44+) and central memory T cells (CD62L+CD44+) in 16‐week‐old Lck‐ECP–transgenic mice (Supplementary Figure 9B). Expression levels of 51 genes, including CD28 and CD69, were significantly up‐regulated (>1.166 fold; *P* < 0.05) in Lck‐ECP–transgenic mouse T cells (Figure 5B). Clusters 2 and 4 were enhanced in Lck‐ECP–transgenic mouse T cells (Figure 5A); both clusters displayed T cell activation and inflammation signatures (Supplementary Figures 9C and D). KEGG pathway analyses revealed that the 51 genes that were up‐regulated in Lck‐ECP–transgenic mouse T cells belonged to the TNF/NFκB signaling pathway, IL‐2/STAT5 signaling pathway, T cell activation, and inflammatory responses (Supplementary Figure 10, *Arthritis & Rheumatology* website at http://onlinelibrary.wiley.com/doi/10.1002/art.41920/abstract). Moreover, GH was the most up‐regulated gene in Lck‐ECP–transgenic mouse T cells compared to wild‐type mouse T cells (Figures 5B and C).

Expression of multiple inflammation‐related genes, such as TNFAIP3, TNFSF8, S100a6, and IFNγ, was also significantly increased in Lck‐ECP–transgenic mouse T cells (Figures 5B and C). The increased IFNγ expression in Lck‐ECP–transgenic mouse T cells (Figure 5C) was consistent with the increased serum IFNγ levels in Lck‐ECP–transgenic mice and enhanced Th1 differentiation of Lck‐ECP–transgenic mouse T cells (Figure 3C and Supplementary Figure 11, available on the *Arthritis & Rheumatology* website at http://onlinelibrary.wiley.com/doi/10.1002/art.41920/abstract). In vitro Th17 differentiation was also modestly enhanced in Lck‐ECP–transgenic mouse T cells (Supplementary Figure 11). Interestingly, Lck‐ECP–transgenic mouse T cells displayed significant induction of Tintin (Figures 5B and C), an intrasarcomeric filamentous protein, which was also identified in T cell–derived CD9+ exosomes from SLE patients by proteomics (Supplementary Table 2). These results suggest that ECP overexpression in T cells induces T cell activation and inflammatory responses.

### Increased numbers of follicular helper T (Tfh) cells and plasma B cells, and increased autoantibody levels, in Lck‐ECP–transgenic mice

Lck‐ECP–transgenic mouse T cells showed significant induction of mRNA for IL‐21, CXCR5, and inducible costimulator (Figure 5D), which are cell markers for Tfh cells. It is notable that programmed death 1 (PD‐1) mRNA levels were not significantly increased in Lck‐ECP T cells (Figure 5D).

**Figure 6:**
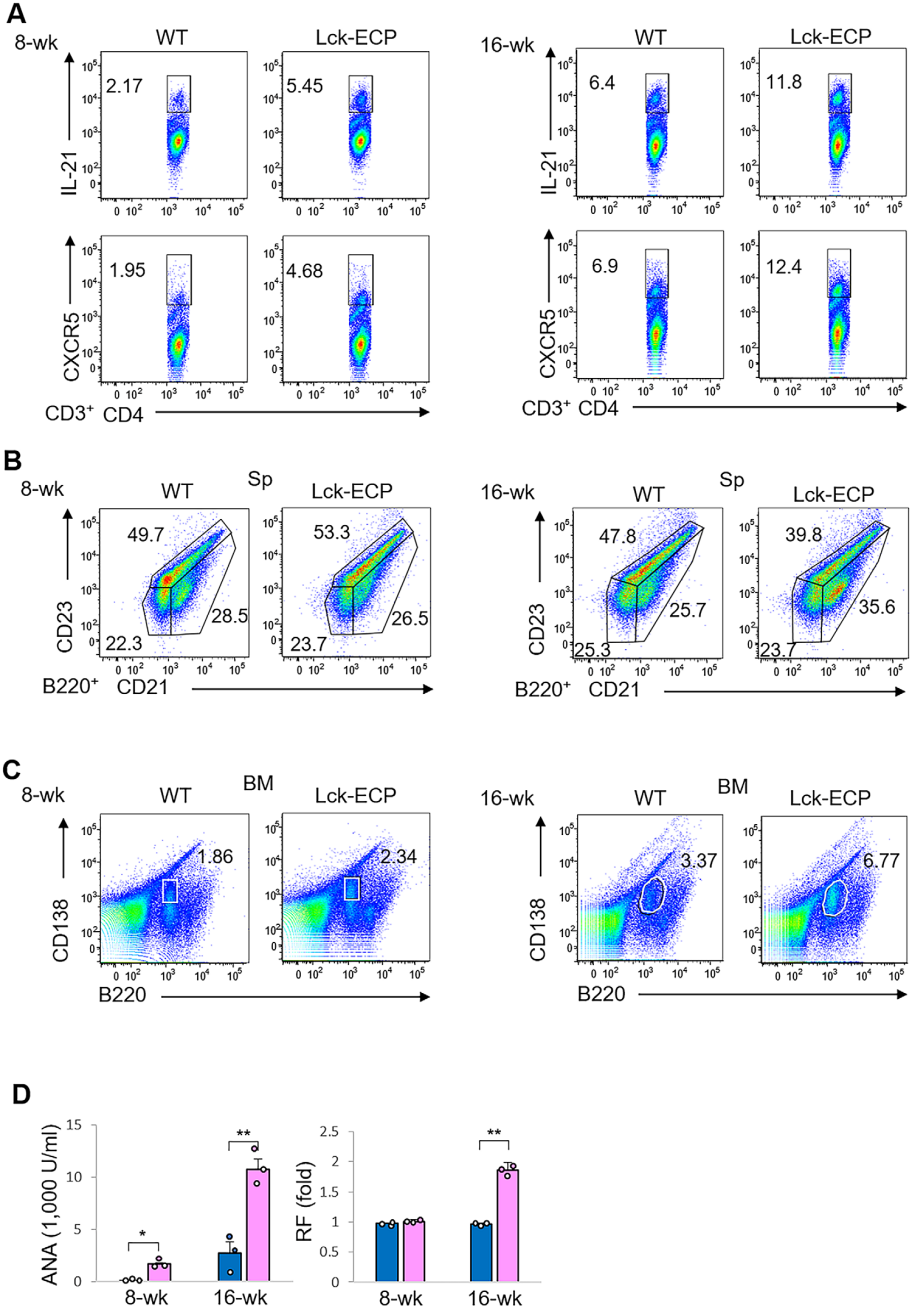
Increase in follicular helper T (Tfh) cell and plasma B cells numbers, and autoantibody levels, in Lck‐ECP–transgenic mice. **A**, Flow cytometric analysis of Tfh (CD3+CD4+IL‐21+ or CD3+CD4+CXCR5+) cells in the spleens (Sp) of 8‐week‐old and 16‐week‐old WT and Lck‐ECP–transgenic mice. **B**, Flow cytometric analysis of B220+ B cells (B220+CD21+/mature B or B220+CD23+/naive B cells) in the spleens of 8‐week‐old and 16‐week‐old WT and Lck‐ECP–transgenic mice. **C**, Flow cytometric analysis of plasma B (B220+CD138+) cells in the bone marrow (BM) of 8‐week‐old and 16‐week‐old WT and Lck‐ECP–transgenic mice. Values next to the outlined areas in **A–C** are the number of positive cells. **D**, Serum antinuclear antibody (ANA) and rheumatoid factor (RF) levels in 8‐week‐old and 16‐week‐old WT mice (blue) and Lck‐ECP–transgenic mice (pink), determined by enzyme‐linked immunosorbent assay. Each symbol represents an individual mouse; bars show the mean ± SEM. * = *P* < 0.05; ** = *P* < 0.01, by Student's 2‐tailed *t*‐test. See Figure 3 for other definitions.

To study whether the Tfh cell population or plasma B cell population was increased in Lck‐ECP–transgenic mice, murine spleen cells and bone marrow cells were analyzed by flow cytometry. We found that the percentages of both CD4+IL‐21+ T cells and CD4+CXCR5+ T cells were increased in 8‐week‐old Lck‐ECP–transgenic mice compared to wild‐type mice (Figure 6A); these 2 T cell subpopulations were further enhanced in 16‐week‐old mice (Figure 6A). Moreover, the percentage of mature B cells (B220+CD21+) was increased, while the percentage of naive B cells (B220+CD23+) was decreased, in the spleens of 16‐week‐old but not 8‐week‐old Lck‐ECP–transgenic mice, compared to wild‐type mice (Figure 6B). The percentage of plasma B cells (B220^low^CD138+) was modestly increased in the bone marrow of 8‐week‐old Lck‐ECP–transgenic mice and was further enhanced in 16‐week‐old Lck‐ECP–transgenic mice (Figure 6C).

Histologic analysis also showed an increased number of germinal centers in 16‐week‐old Lck‐ECP–transgenic mice (Supplementary Figure 12, available on the *Arthritis & Rheumatology* website at http://onlinelibrary.wiley.com/doi/10.1002/art.41920/abstract). Consistent with these findings, serum levels of ANA were slightly induced in 8‐week‐old Lck‐ECP–transgenic mice and further increased in 16‐week‐old Lck‐ECP–transgenic mice (Figure 6D). Serum levels of RF were also increased in 16‐week‐old, but not 8‐week‐old, Lck‐ECP–transgenic mice compared to wild‐type mice (Figure 6D). These findings suggest that induction of Tfh cells in Lck‐ECP–transgenic mice results in an increase in the number of plasma B cells, facilitating subsequent autoantibody production.

## DISCUSSION

A key finding of this study was the identification of one novel T cell exosomal protein, ECP, which plays an important role in SLE pathogenesis. ECP overexpression in T cells resulted in enhancement of inflammatory responses and T cell activation. Notably, ECP‐containing exosomes from T cells targeted several tissues (such as the liver, kidney, and joint) of the recipient mice, leading to tissue inflammation. These data suggest that ECP‐overexpressing T cells or ECP‐containing exosomes may act as a causal factor in SLE.

One of the notable findings of this study is that T cell–derived exosomal ECP contributes to autoimmune diseases. This is the first study to show that ECP is an exosomal protein. Extracellular ECP stimulation using ECP recombinant protein induces cell death, including necrosis and apoptosis (29, 31, 32); however, T cell development in Lck‐ECP–transgenic mice was not affected. The data suggest that ECP recombinant protein acts differently from exosomal and intracellular ECP. In support of this notion, soluble ECP levels were not increased in the sera of human SLE patients. These findings also suggest that exosomal ECP and ECP‐overexpressing T cells contribute to autoimmune responses through cell death–independent pathways. Furthermore, adoptive transfer of ECP‐containing exosomes induced autoantibody production and inflammation expansion; it is likely that the T cell–derived exosomes from Lck‐ECP–transgenic mouse T cells contain other inflammatory molecules in addition to ECP proteins.

Besides induction of the proinflammatory cytokine IFNγ, Lck‐ECP–transgenic mice manifested the induction of Tfh cells, plasma B cells, and autoantibodies. These results suggest that ECP overexpression in T cells induces the Tfh cell population, facilitating plasma B cell differentiation, leading to overproduction of autoantibodies. Besides induction of plasma B cells through Tfh cells, it is also possible that secreted molecules from ECP‐overexpressing T cells or other proteins within ECP+ exosomes may stimulate/activate other potential target cells (e.g., B cells, macrophages, dendritic cells, or osteoclasts), leading to multiple inflammatory phenotypes.

The aforementioned findings are consistent with our single‐cell RNA‐sequencing data using Lck‐ECP–transgenic mouse T cells. First, Lck‐ECP–transgenic mouse T cells showed highly increased levels of GH, which induces T cell survival and activation (37, 38). Consistent with these findings, T cell activation, T cell proliferation, and adaptive immune response pathways were indeed induced in Lck‐ECP–transgenic mouse T cells. Lck‐ECP–transgenic mouse T cells also showed induction of IFNγ+ Th1 differentiation.

Second, ECP signaling induced several proinflammatory cytokines/chemokines, including TNFSF8 (also called CD30 ligand [CD30L]), S100 proteins, and IFNγ, that may contribute to inflammation of multiple tissue types. CD30L up‐regulation is involved in the pathogenesis of human SLE, RA, Hodgkin lymphoma, and anaplastic large cell lymphoma (39). S100 protein enhancement contributes to arthritis and neural degenerative diseases (40, 41). Chronic IFNγ overproduction induces hepatoxicity (42, 43); IFNγ also plays a crucial role in the development of nephritis (44, 45). Interestingly, our ELISA data also showed early induction of IFNγ in Lck‐ECP–transgenic mice. These previous publications and our data suggest that ECP‐overexpressing T cells and ECP‐containing exosomes cooperate with the aforementioned proinflammatory cytokines/chemokines to induce nephritis, arthritis, and hepatitis in Lck‐ECP–transgenic mice and maybe also in human SLE patients.

Third, surface receptors on exosomes may determine their tissue tropisms. It would be interesting to study whether the exosomal olfactory receptor 7D2, identified in this study as being enriched in SLE (Supplementary Table 2), controls the tissue tropism of inflammatory T cell exosomes. Finally, the role of ECP‐inducible Titin, an intrasarcomeric filamentous protein, in SLE pathogenesis needs to be explored.

Taken together, these findings indicate that ECP overexpression in T cells contributes to inflammatory responses through both intrinsic and extrinsic events. To distinguish the specific role of ECP in T cells versus exosomes is highly challenging, if not impossible, due to the following two reasons. First, there is no definitive ECP orthologous gene (46) for the generation of ECP‐knockout mice. Second, there are no exosome‐specific surface markers for the depletion of exosomes in vivo.

SLE patients experience damage to multiple organs and complex symptoms (1). Understanding the causal factors of individual symptoms will help in the development of novel therapeutics for SLE. In this study, we found that ECP‐containing exosomes induce arthritis, hepatitis, and nephritis in mice. According to our clinical data and proteomics analysis, 5 SLE patients had ECP‐containing exosomes derived from T cells, while all of these 5 SLE patients developed arthritis. The data suggest that the novel pathogenic factor, ECP‐containing exosome, may also be a biomarker for SLE‐associated arthritis. In addition, 2 of these 5 SLE patients have developed nephritis; however, it is difficult to diagnosis SLE‐associated nephritis at an early stage. ECP‐containing exosomes may help in the early diagnosis of SLE‐associated nephritis. Although up to 50% of SLE patients develop hepatitis, the exact diagnosis of hepatitis remains challenging due to complex conditions, including infection, drug treatment, or SLE (47). Notably, Lck‐ECP–transgenic mice spontaneously developed hepatitis, suggesting that hepatitis in SLE patients may be a consequence of the induction of ECP‐containing exosomes. Thus, exosomal ECP may also be a potential biomarker for SLE‐associated hepatitis.

Taken together, our findings suggest that ECP overexpression in T cells or T cell–derived exosomes induces T cell hyperactivation and proinflammatory cytokine production through both intrinsic and extrinsic events, leading to inflammation in multiple organs and autoimmune responses. Thus, ECP‐overexpressing T cells and ECP‐containing exosomes are potential biomarkers for SLE.

## AUTHOR CONTRIBUTIONS

All authors were involved in drafting the article or revising it critically for important intellectual content, and all authors approved the final version to be published. Dr. Tan had full access to all of the data in the study and takes responsibility for the integrity of the data and the accuracy of the data analysis.

### Study conception and design

Chuang, Tan.

### Acquisition of data

Chuang, M. Chen, Y. Chen, Ciou, Hsueh, Tsai.

### Analysis and interpretation of data

Chuang, M. Chen, Y. Chen, Ciou, Hsueh, Tsai, Tan.

## Supporting information


**Appendix** S1. Supporting Information

Disclosure Form
