# Supplementary material for: Induction of Interferon‐γ and Tissue Inflammation by Overexpression of Eosinophil Cationic Protein in T Cells and Exosomes
Source: Arthritis Rheumatol. 2021 Dec 9;74(1):92–104. doi: 10.1002/art.41920 (PMC9300123; doi:10.1002/art.41920)
Supplement: Supplementary file 1 — Appendix S1. Supporting Information [file ART-74-92-s001.pdf]

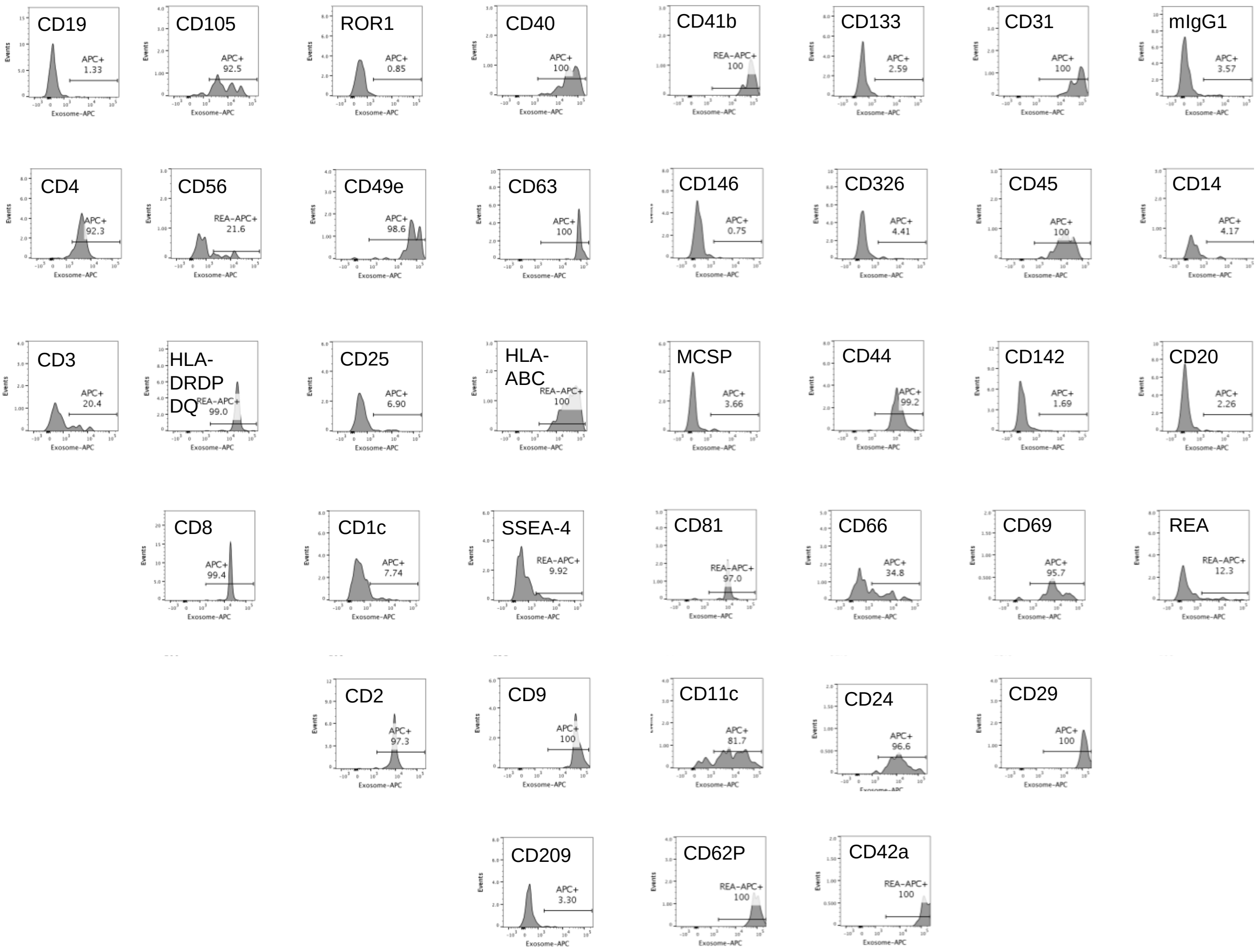

Supplementary Figure 1. Individual surface proteins on T-cell-derived exosomes are characterized by MACSPlex exosome analysis. Flow cytometry analysis of 37 individual surface proteins on exosomes isolated from T-cell supernatants of 12 SLE patients and 12 healthy controls. Data show individual percentages of exosomes within FITC/PE-labelled beads conjugated with APC-labeled antibodies against surface proteins. Histograms from a representative SLE patient are shown.

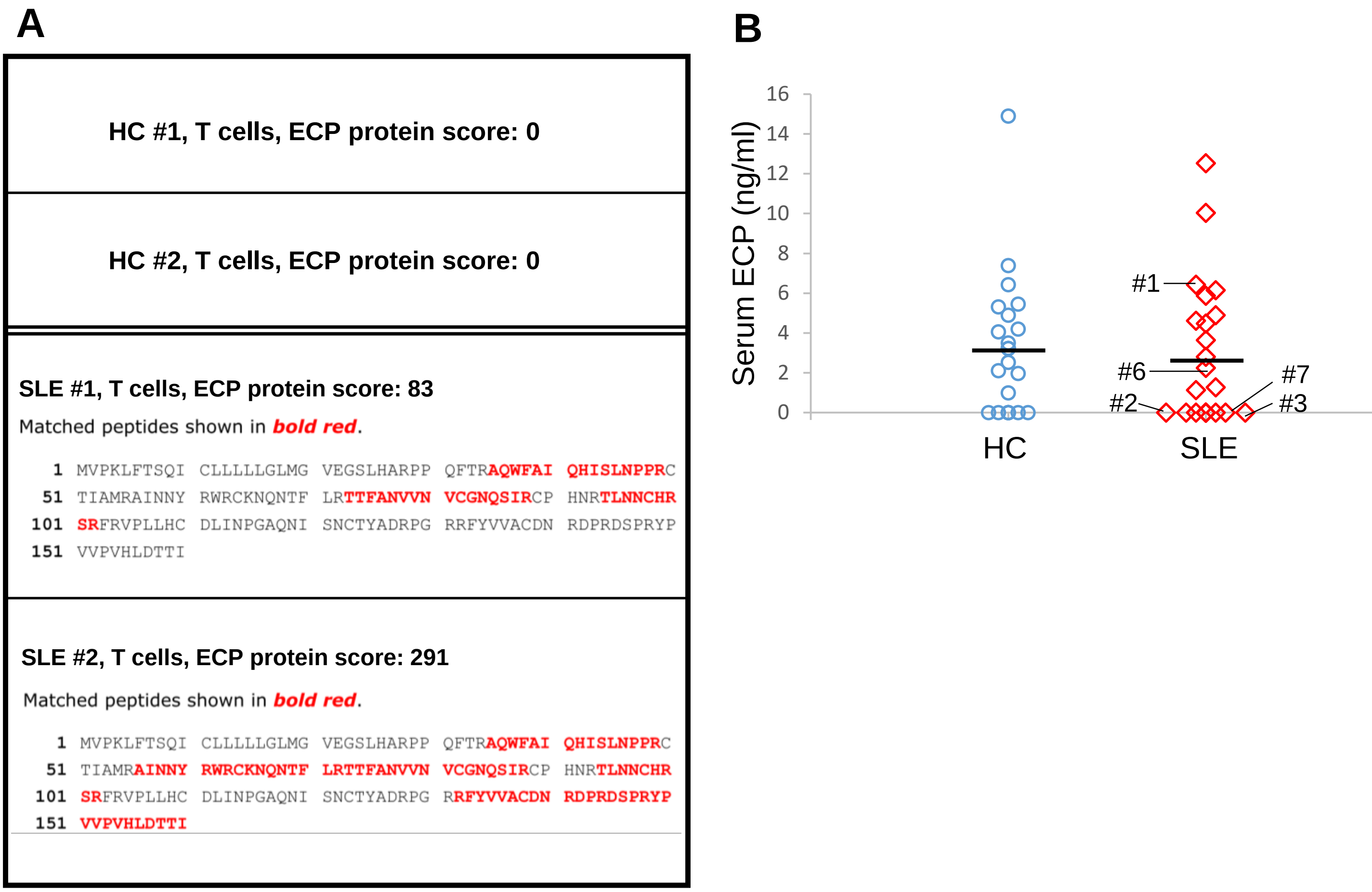

Supplementary Figure 2. ECP is induced in peripheral blood T cells of SLE patients. (**A**) Identification of ECP by mass spectrometry-based protein sequencing of peripheral blood T cells from 2 SLE patients (#1 and #2). ECP was not detected in T cells from 2 healthy controls (HC, #1 and #2). The protein score is the sum of the highest ions score of MS/MS search for each distinct peptide. (**B**) Soluble ECP levels in the sera from 24 SLE patients (including #1, #2, #3, #6, #7 used for exosome proteomics) and 24 healthy controls were measured by ELISA.

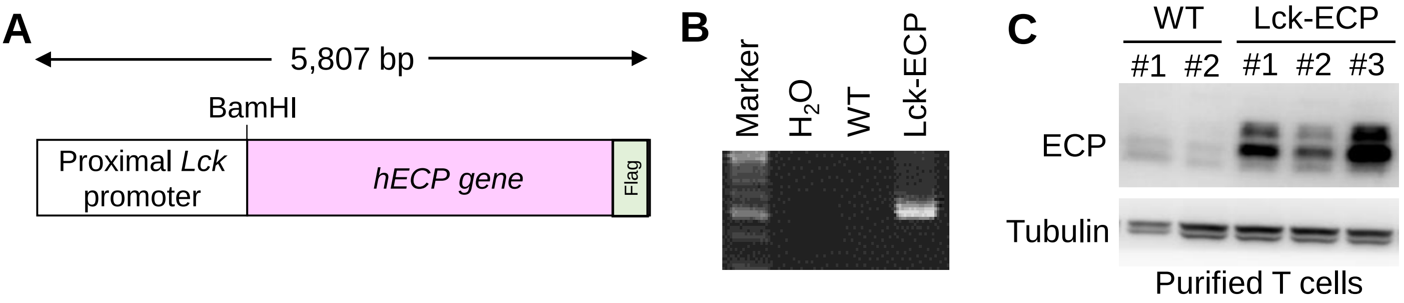

Supplementary Figure 3. Generation of T-cell-specific human ECP transgenic mice. (**A**) Schematic diagram for the construction of the Lck promoter-driven human ECP plus a Flag tag. (**B**) Genotyping of T-cell-specific ECP transgenic (Lck-ECP) mice and wild-type (WT) mice. (**C**) Immunoblotting analysis of transgenic human ECP protein levels in splenic T cells from 3 Lck-ECP Tg and 2 WT mice.

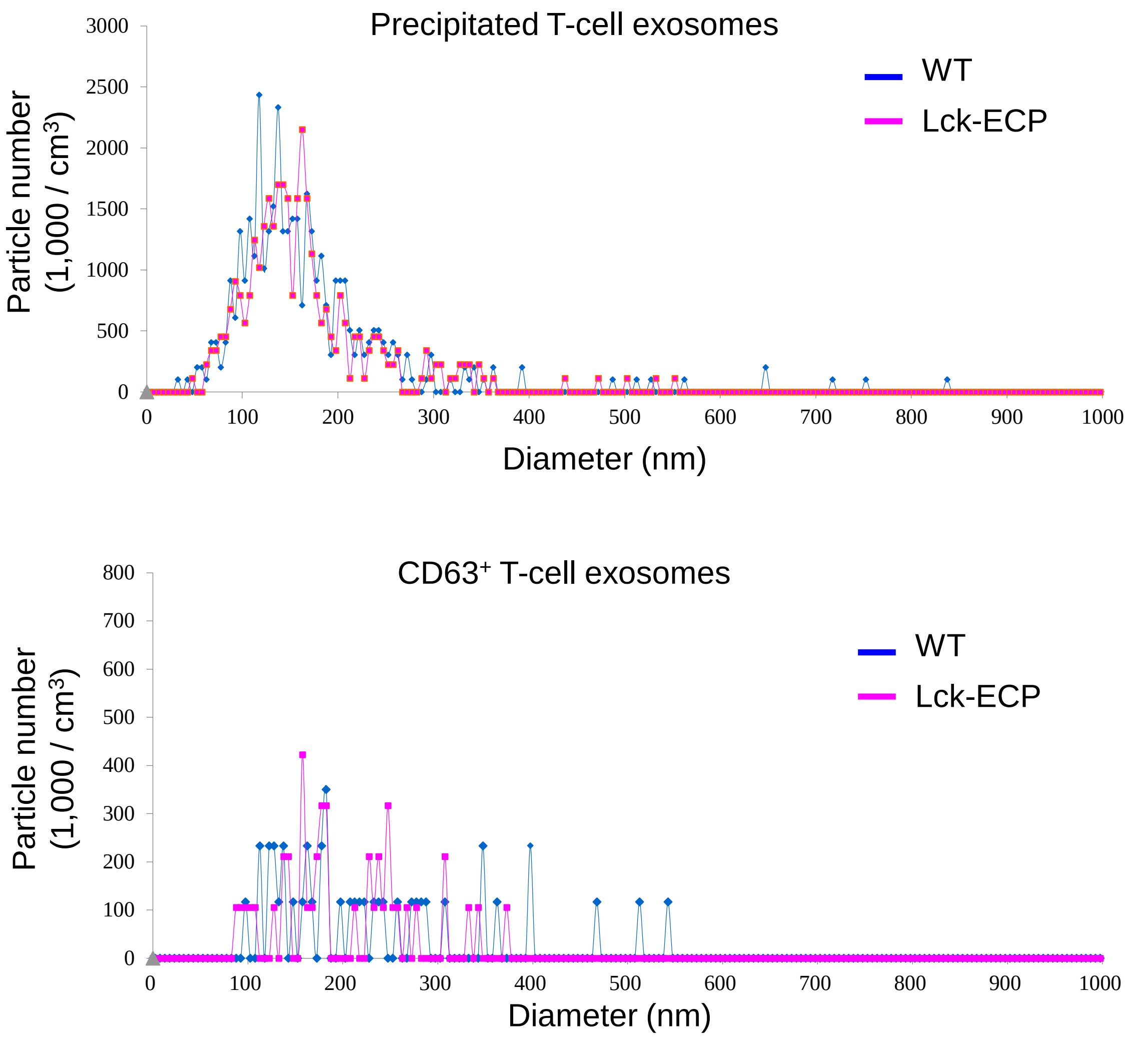

Supplementary Figure 4. The numbers and sizes of extracellular vesicles from Lck-ECP transgenic T cells are comparable to wild-type T cells. (**A**) ZetaView analysis of particle numbers and sizes of precipitated extracellular vesicles in supernatants from Lck-ECP Tg and wild-type (WT) T cells. Extracellular vesicles were precipitated by ExoQuick-TC. (**B**) ZetaView analysis of particle numbers and sizes of CD63^{+} extracellular vesicles in supernatants from Lck-ECP Tg and wild-type (WT) T cells. Extracellular vesicles were precipitated by ExoQuick-TC, followed by resuspension. CD63^{+} extracellular vesicles were further immunoprecipitated by anti-CD63 antibody-conjugated beads.

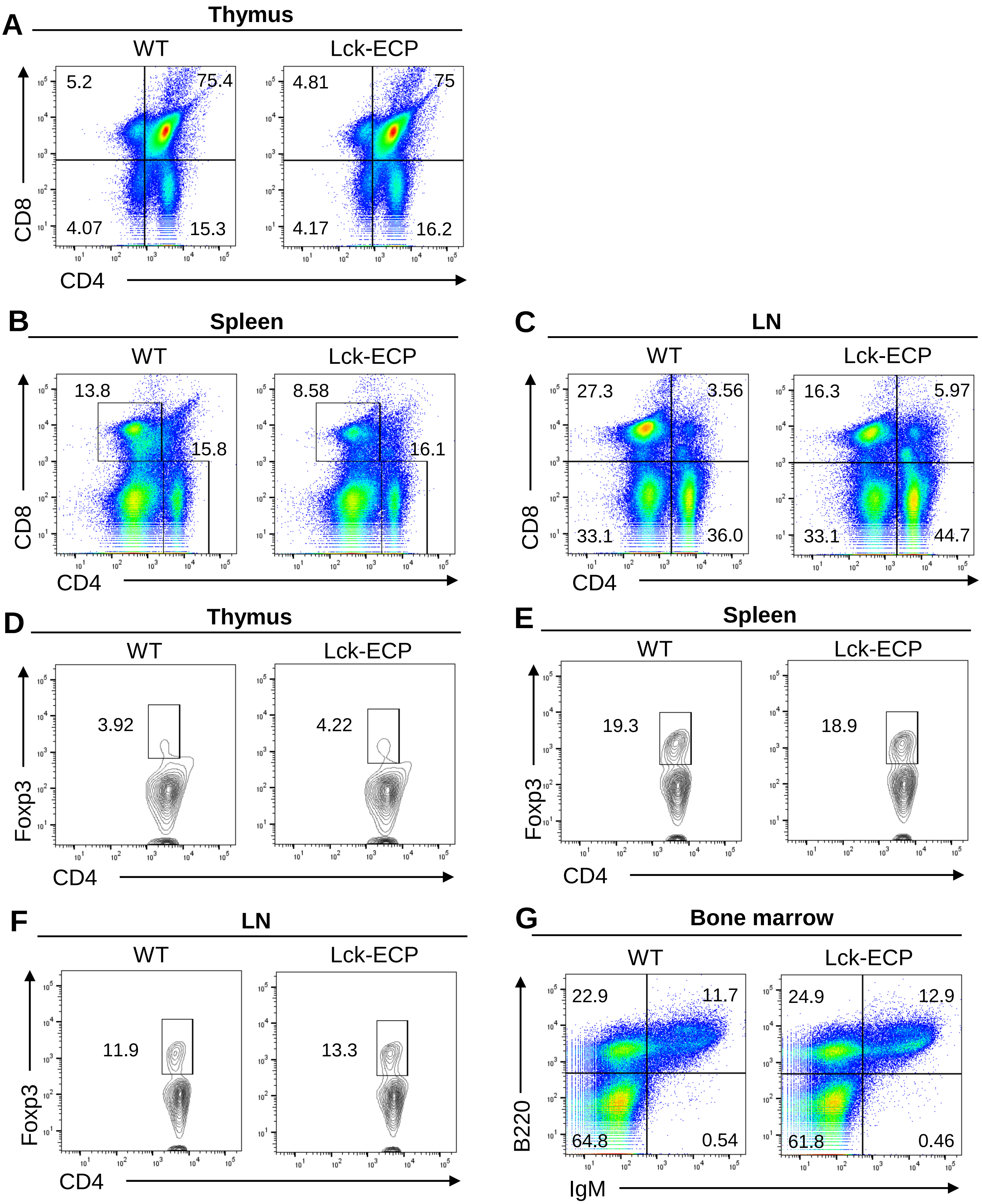

Supplementary Figure 5. Normal T-cell and B-cell development in thymus and bone marrow of Lck-ECP transgenic mice. (**A-E**) Flow cytometry analyses of T cells (A-C) and Treg cells (D-F) from the thymus, spleen, or lymph nodes of 5-week-old wild-type (WT) or Lck-ECP transgenic mice. (**G**) Flow cytometry analyses of $B220^+$ B cells from the bone marrow of WT or Lck-ECP transgenic mice. Data shown are representatives of three independent experiments.

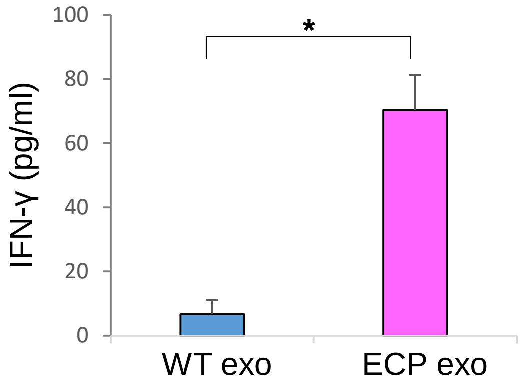

Supplementary Figure 6. Adoptive transfer of ECP exosomes induces serum IFN-γ levels in wild-type recipient mice. Exosomes derived from wild-type or Lck-ECP T cells (WT exo or ECP exo) were adoptively transferred into recipient mice by intravenous injection every 3 days for 30 days. n = 3 per group. ELISA of serum IFN-γ levels in recipient mice. *, P value < 0.05 (two-tailed Student's t-test).

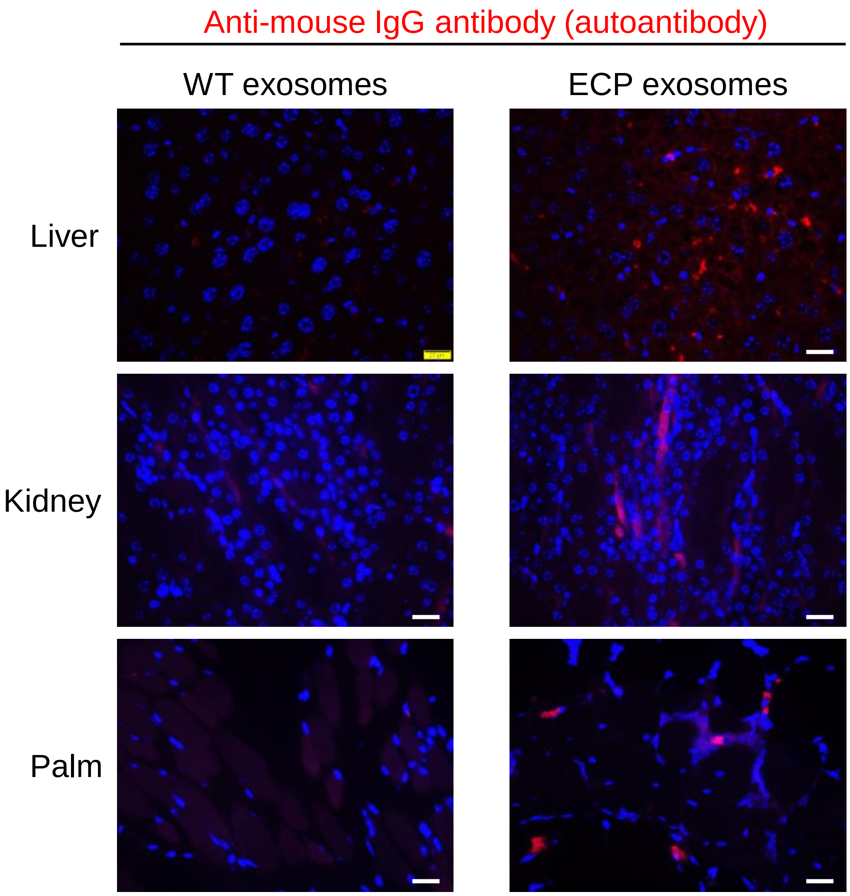

Supplementary Figure 7. Adoptive transfer of ECP exosomes induces autoantibody deposition in tissues of the recipient mice. Exosomes derived from wild-type or Lck-ECP T cells (WT exo or ECP exo) were adoptively transferred into the wild-type recipient mice by intravenous injection every 3 days for 30 days. n = 3 per group. Immunohistochemical staining of Alexa 647-conjugated anti-mouse IgG antibody (red) in the paraffin-embedded sections of the liver, kidney, and Palm from recipient mice. Cell nucleus was stained with DAPI (blue). Scale bars, 20 μm.

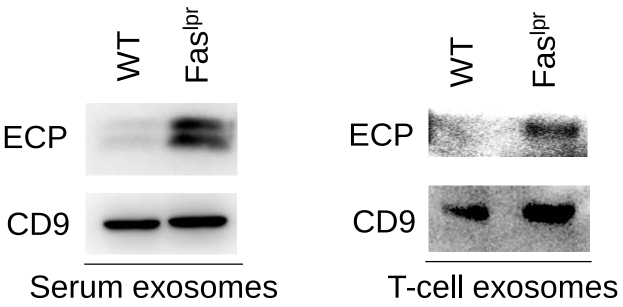

Supplementary Figure 8. Exosomal ECP levels are increased in MRL/MpJ-Fas^{lpr} autoimmune lupus model mice. Immunoblotting analysis of ECP and CD9 protein levels in serum exosomes (left panel) and T-cell-derived exosomes (right panel) from MRL/MpJ-Fas^{lpr} and MRL/MpJ wild-type mice.

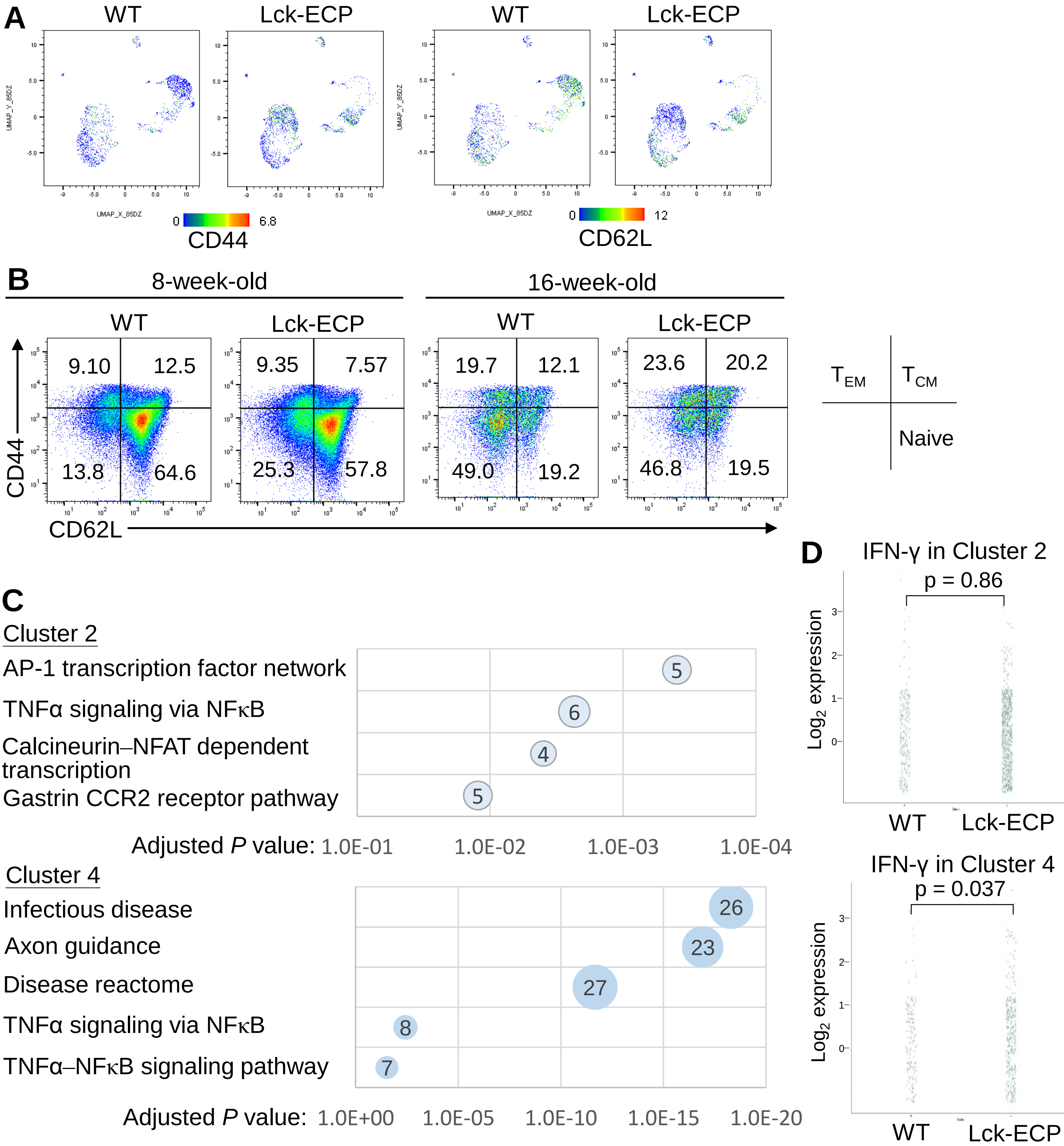

Supplementary Figure 9. Memory Th1 cells and effector Tc cells are increased in Lck-ECP transgenic mice. (**A**) Single-cell gene expression of CD44 (left panel) and CD62L (right panel) in WT and Lck-ECP transgenic T cells. Naïve T cells, $CD44^{low}CD62L^{high}$; effector T cells, $CD44^{high}CD62L^{low}$; memory T cells, $CD44^{high}CD62L^{high}$ cells. (**B**) Flow cytometry analyses of naïve T, central memory T (T_{CM}), and effector memory T (T_{EM}) cells from the spleen of 8-week-old and 16-week-old wild-type (WT) or Lck-ECP transgenic mice. (**C**) KEGG pathway enrichment of Cluster 2 (Memory Th1 cells) and Cluster 4 (effector Tc cells) in Lck-ECP transgenic T cells. Pathways belonging to different classifications are listed on the left of the plot. Varied numbers of genes enriched in individual pathways are presented by different diameter sizes and numbers for individual dots. (**D**) Jitter plots for the expression of IFN-γ in Cluster 2 and Cluster 4 of WT and Lck-ECP transgenic T cells.

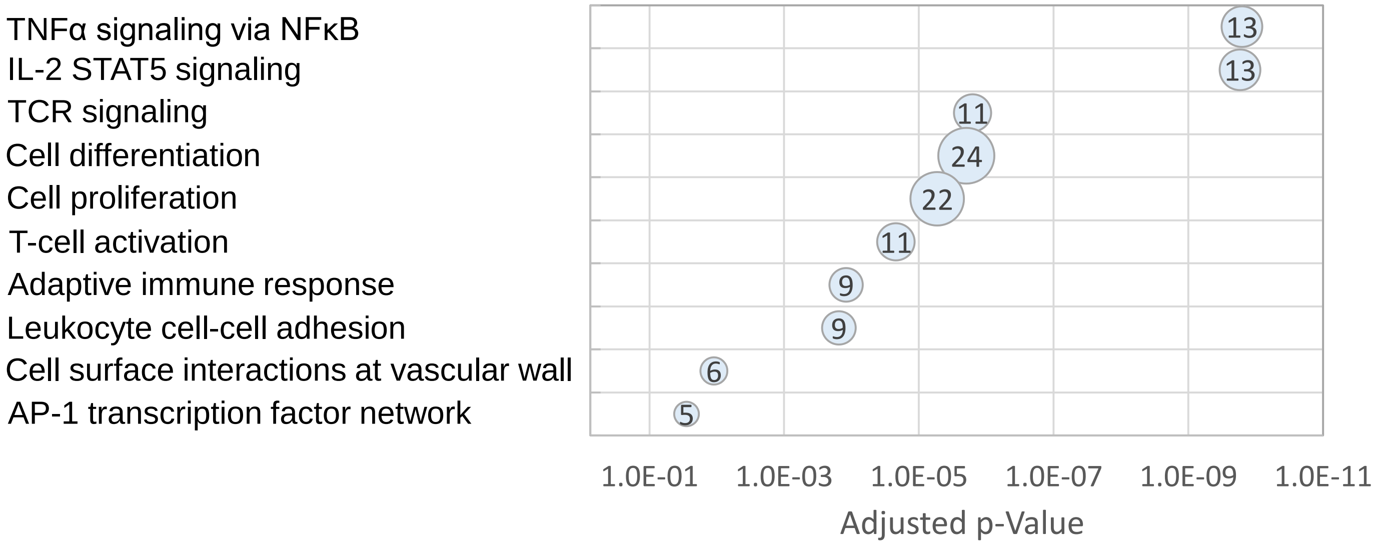

Supplementary Figure 10. ECP overexpression in T cells induces TNF-α signaling, IL-2 signaling, and T-cell activation pathways. KEGG (Kyoto Encyclopedia of Genes and Genomes) pathway enrichment of upregulated genes in Lck-ECP transgenic T cells. Pathways belonging to different classifications are listed on the left of the plot. Varied numbers of genes enriched in individual pathways are presented by different diameter sizes and numbers for individual dots.

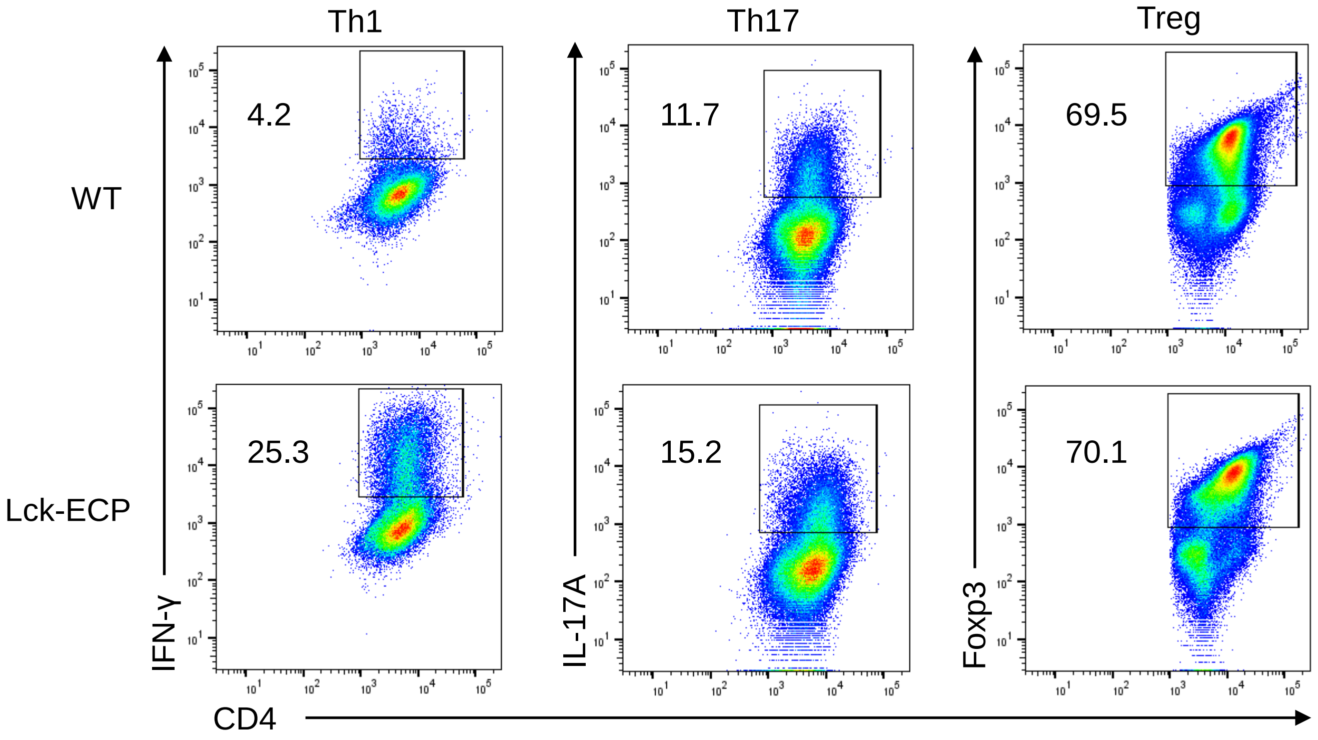

Supplementary Figure 11. Th1 differentiation is enhanced by ECP transgene. Flow cytometry analyses of IFN-γ-producing, IL-17A-producing, and Foxp3-positive $CD4^+$ T cells. Data are representative of at least three independent experiments.

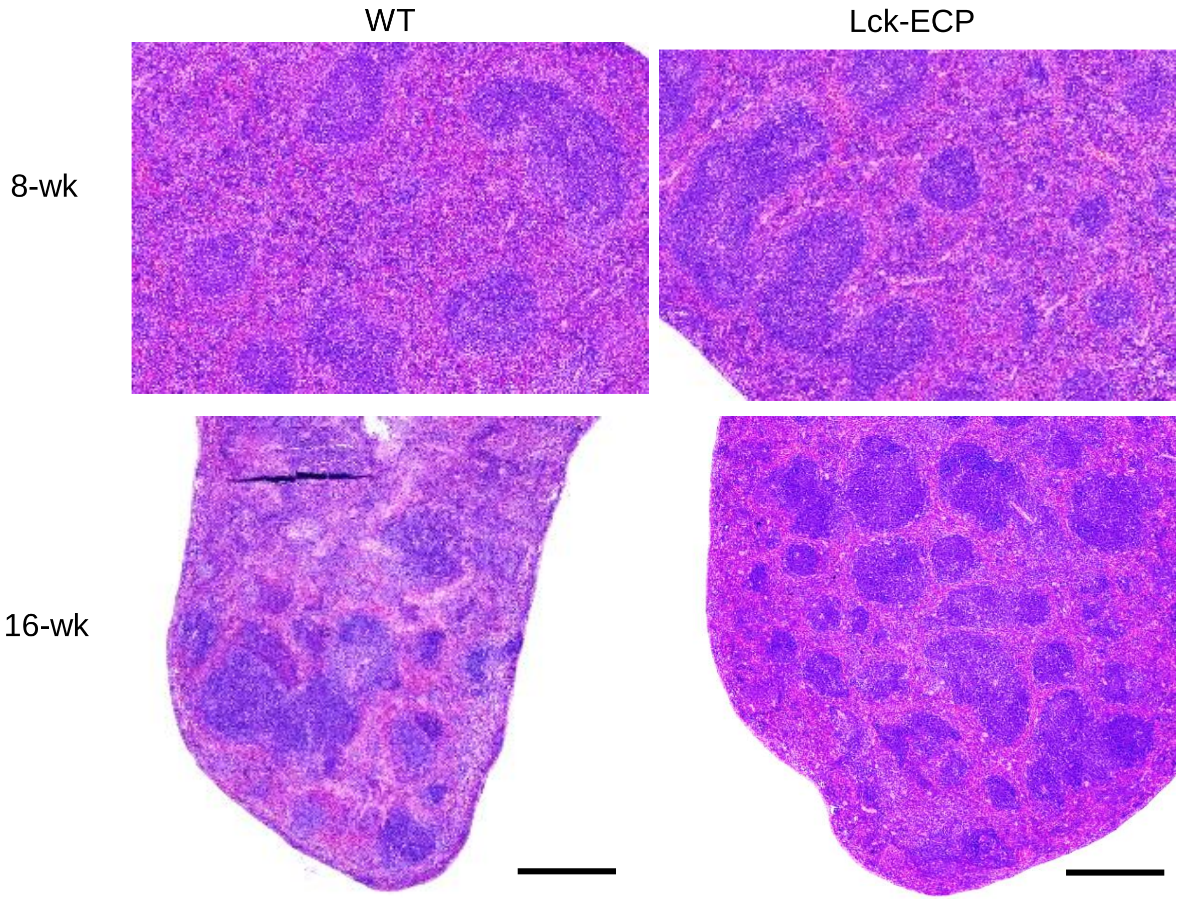

Supplementary Figure 12. The number of the germinal center is increased in aged Lck-ECP transgenic mice. Hematoxylin and eosin (H&E)–stained sections of the spleen from 8-week old and 16-week-old wild-type or Lck-ECP transgenic mice. Scale bars, 500 µm.

Supplementary Table 1. Profile of enrolled SLE patients

SLE#	Gender	Age	SLEDAI	Duration (year)	dsDNA (U/ml)	C3 (mg/dl)	C4 (mg/dl)	WBC (/mm^3)	PLT (1,000/mm^3)	HgB (g/dl)	Arthritis	Nephritis	Hepatitis§	Cohort	T cell-exosome	
															MACSPlex	Proteomics
1	F	40	6	13	53.5	64.8	19.5	9250	402	12.1	1	1	1	1		✓
2	F	21	4	1	62.4	61.6	14.2	4210	317	12.8	1	0	0	1		✓
3	F	27	6	3	11.8	71.1	19.2	8600	252	12.9	1	0	0	1		✓
4	F	66	2	46	76.0	88.5	17.5	6600	150	13.9	1	1	1	2	✓	
5	F	26	5	13	15.6	68.6	11.6	2700	213	11.1	1	0	1	2	✓	
6	F	66	10	0.2*	114.0	56.8	6.6	3400	203	10	1	0	1	2		✓
7	F	42	2	0.3*	0	128.0	33.7	12,000	262	14.1	1	0	1	2		✓
8	F	47	4	1	40.9	81.4	7.1	6700	356	12.4	1	0	1	2	✓	
9	F	45	16	12	221.4	88.4	10.2	5110	303	11.7	0	0	1	1	✓	
10	F	54	10	0.3*	212.7	89.7	20.1	4190	278	9.9	1	1	1	1	✓	
11	M	27	6	9	67.8	103.6	15.9	9040	226	14.7	1	0	1	1	✓	
12	F	39	2	0.3*	57.4	65.2	3.9	7380	137	12.2	0	0	1	1	✓	
13	F	43	2	9	11.4	92.3	13.7	10,100	31	14.6	0	1	1	2	✓	
14	F	22	36	0*	88.5	21.5	2.3	7500	97	9.8	1	1	0	2	✓	
15	F	35	4	5	24.8	110.0	27.0	8800	224	13.3	1	1	0	2	✓	
16	F	34	15	5	23.0	38.4	6.7	5900	253	8.3	0	1	1	2	✓	
17	F	33	15	0*	247.0	38.4	6.7	5900	172	8.3	1	1	1	2	✓	
18	F	63	5	15	575.3	79.9	15.6	3330	196	12.9	1	1	1	1		
19	F	20	12	2.5	49.6	99.5	15.0	3670	205	12.2	1	1	0	1		
20	F	21	4	7	20.2	70.6	8.7	4300	323	10.3	0	0	0	2		
21	F	35	4	4	83.8	71.8	10.3	2400	183	11.1	0	0	0	2		
22	F	43	1	9	11.4	13.7	13.7	10100	317	14.6	0	1	1	2		
23	F	22	32	0*	88.5	21.5	2.3	7500	97	9.8	1	1	0	2		
24	F	33	20	0*	247	21.1	1.3	5200	172	8.3	1	1	1	2		

Cohort 1, patients from the Division of Immunology and Rheumatology at Taichung Veterans General Hospital in Taiwan
Cohort 2, patients from the Division of Immunology and Rheumatology at Taipei Veterans General Hospital in Taiwan
*, newly diagnosed patient; §, patients with hepatitis during 2018-2020.
SLEDAI, SLE disease activity index; C3, complement C3; C4, complement C4; PLT, platelet; HgB, haemoglobin.

Supplementary Table 2. SLE-enriched exosomal proteins of CD9$^+$ or CD63$^+$ T-cell exosomes

Proteomics Score of SLE-enriched exosomal proteins / Protein score	SLE #1		SLE #2		SLE #3		SLE #6		SLE #7	
	CD9$^+$	CD63$^+$	CD9$^+$	CD63$^+$	CD9$^+$	CD63$^+$	CD9$^+$	CD63$^+$	CD9$^+$	CD63$^+$
Eosinophil cationic protein (ECP)	70	0	167	210	0	56	117	200	541	462
Bactericidal/permeability-increasing protein (BPI)	78	0	0	36	127	262	0	182	1416	1393
Hemoglobin subunit beta (HBB)	13	24	21	20	0	0	171	78	67	68
Collagen alpha-1(I) chain (COL1A1)	31	25	0	35	27	0	44	0	62	0
Protein cramped-like (CRAMP1)	67	62	60	55	0	0	0	0	0	0
Dual specificity testis-specific protein kinase 2 (TESK2)	30	25	30	49	0	0	0	0	0	0
Integrator complex subunit 9 (INTS9)	27	26	28	28	0	0	0	0	0	0
DNA topoisomerase 1 (TOP1)	29	37	29	29	0	0	0	0	0	0
WD repeat and HMG-box DNA-binding protein 1 (WDHD1)	19	18	19	20	0	0	0	0	0	0
DNA repair protein REV1 (REV1)	25	18	18	0	0	0	0	0	0	0
NGFI-A-binding protein 1 (NAB1)	0	0	20	0	0	0	0	0	0	0
Klotho (KL)	17	0	17	0	0	0	0	0	0	0
Probable G-protein coupled receptor 156 (GPR156)	17	23	17	17	0	0	0	0	0	0
Glutamine-rich protein 2 (QRICH2)	17	17	17	16	0	0	0	0	0	0
Titin (TTN)	16	22	18	20	0	0	0	0	0	0
Selenium-binding protein 1 (SELENBP1)	16	15	18	48	0	0	0	0	0	0

Ankyrin repeat and BTB/POZ domain-containing protein 2 (ABTB2)	15	0	20	14	0	0	0	0	0	0
Androglobin (ADGB)	15	19	19	19	0	0	0	0	0	0
Collagen alpha-1(III) chain (COL3A1)	0	24	32	35	0	0	0	0	0	0
Histone-lysine N-methyltransferase SMYD1 (SMYD1)	19	20	0	25	0	0	0	0	0	0
Zinc finger protein 510 (ZNF510)	0	26	19	24	0	0	0	0	0	0
WD repeat and HMG-box DNA-binding protein 1 (WDHD1)	19	18	19	20	0	0	0	0	0	0
COMM domain-containing protein 5 (COMMD5)	0	22	0	19	0	0	0	0	0	0
Olfactory receptor 7D2 (OR7D2)	0	17	0	18	0	0	0	0	0	0
Kinesin-like protein KIF26A (KIF26A)	17	20	0	16	0	0	0	0	0	0
Probable tRNA N6-adenosine threonylcarbamoyltransferase (OSGEPL1)	0	20	0	16	0	0	0	0	0	0
Neurofilament medium polypeptide (NEFM)	0	21	0	16	0	0	0	0	0	0
Kelch domain-containing protein 8A (KLHDC8A)	0	17	0	15	0	0	0	0	0	0
MORF4 family-associated protein 1-like 1 (MRFAP1L1)	0	19	15	15	0	0	0	0	0	0
