# Supplementary material for: Induction of Interferon‐γ and Tissue Inflammation by Overexpression of Eosinophil Cationic Protein in T Cells and Exosomes
Source: Arthritis Rheumatol. 2021 Dec 9;74(1):92–104. doi: 10.1002/art.41920 (PMC9300123; doi:10.1002/art.41920)
Supplement: Supplementary file 2 — Disclosure Form [file ART-74-92-s002.pdf]

ICMJE DISCLOSURE FORM

Date:__June 6, 2021 ____
Your Name:__Huai-Chia Chuang ____
Manuscript Title:__ ECP Overexpression in T Cells and Exosomes Induces IFN-γ Production and Tissue Inflammation
Manuscript number (if known):__ ar-20-2148 ____

In the interest of transparency, we ask you to disclose all relationships/activities/interests listed below that are related to the content of your manuscript. "Related" means any relation with for-profit or not-for-profit third parties whose interests may be affected by the content of the manuscript. Disclosure represents a commitment to transparency and does not necessarily indicate a bias. If you are in doubt about whether to list a relationship/activity/interest, it is preferable that you do so.

The following questions apply to the author's relationships/activities/interests as they relate to the current manuscript only.

The author's relationships/activities/interests should be defined broadly. For example, if your manuscript pertains to the epidemiology of hypertension, you should declare all relationships with manufacturers of antihypertensive medication, even if that medication is not mentioned in the manuscript.

In item #1 below, report all support for the work reported in this manuscript without time limit. For all other items, the time frame for disclosure is the past 36 months.

		**Name all entities with whom you have this relationship or indicate none (add rows as needed)**	**Specifications/Comments (e.g., if payments were made to you or to your institution)**
		**Time frame: Since the initial planning of the work**	
1	All support for the present manuscript (e.g., funding, provision of study materials, medical writing, article processing charges, etc.) **No time limit for this item.**	_ X _ None	
		**Time frame: past 36 months**	
2	Grants or contracts from any entity (if not indicated in item #1 above).	___ None	
		Grants from Ministry of Science and Technology, Taiwan	109-2326-B-400-005-MY3, 108-2628-B-400-001, 108-2314-B-400-015
3	Royalties or licenses	__ X __ None	

4	Consulting fees	__ X __ None	
5	Payment or honoraria for lectures, presentations, speakers bureaus, manuscript writing or educational events	__ X __ None	
6	Payment for expert testimony	__ X __ None	
7	Support for attending meetings and/or travel	__ X __ None	
8	Patents planned, issued or pending	__ X __ None	
9	Participation on a Data Safety Monitoring Board or Advisory Board	__ X __ None	
10	Leadership or fiduciary role in other board, society, committee or advocacy group, paid or unpaid	__ X __ None	
11	Stock or stock options	__ X __ None	
12	Receipt of equipment, materials, drugs, medical writing, gifts or other services	___X _ None	
13	Other financial or non-financial interests	__ X __ None	

Please place an "X" next to the following statement to indicate your agreement:

_X_ I certify that I have answered every question and have not altered the wording of any of the questions on this form.

ICMJE DISCLOSURE FORM

Date:__June 6, 2021 ____
Your Name:__ Ming-Han Chen ____
Manuscript Title:__ ECP Overexpression in T Cells and Exosomes Induces IFN-γ Production and Tissue Inflammation
Manuscript number (if known):__ ar-20-2148 ____

In the interest of transparency, we ask you to disclose all relationships/activities/interests listed below that are related to the content of your manuscript. "Related" means any relation with for-profit or not-for-profit third parties whose interests may be affected by the content of the manuscript. Disclosure represents a commitment to transparency and does not necessarily indicate a bias. If you are in doubt about whether to list a relationship/activity/interest, it is preferable that you do so.

The following questions apply to the author's relationships/activities/interests as they relate to the current manuscript only.

The author's relationships/activities/interests should be defined broadly. For example, if your manuscript pertains to the epidemiology of hypertension, you should declare all relationships with manufacturers of antihypertensive medication, even if that medication is not mentioned in the manuscript.

In item #1 below, report all support for the work reported in this manuscript without time limit. For all other items, the time frame for disclosure is the past 36 months.

		**Name all entities with whom you have this relationship or indicate none (add rows as needed)**	**Specifications/Comments (e.g., if payments were made to you or to your institution)**
**Time frame: Since the initial planning of the work**			
1	All support for the present manuscript (e.g., funding, provision of study materials, medical writing, article processing charges, etc.) **No time limit for this item.**	_X_ None	
**Time frame: past 36 months**			
2	Grants or contracts from any entity (if not indicated in item #1 above).	___ None	
		Grants from ministry of science and technology, Taiwan	108-2314-B-075-019
		Grants from ministry of science and technology, Taiwan	109-2314-B-075 -085

3	Royalties or licenses	__ X __ None	
4	Consulting fees	__ X __ None	
5	Payment or honoraria for lectures, presentations, speakers bureaus, manuscript writing or educational events	__ X __ None	
6	Payment for expert testimony	__ X __ None	
7	Support for attending meetings and/or travel	__ X __ None	
8	Patents planned, issued or pending	__ X __ None	
9	Participation on a Data Safety Monitoring Board or Advisory Board	__ X __ None	
10	Leadership or fiduciary role in other board, society, committee or advocacy group, paid or unpaid	__ X __ None	
11	Stock or stock options	__ X __ None	
12	Receipt of equipment, materials, drugs, medical writing, gifts or other services	___X _ None	
13	Other financial or non-financial interests	__ X __ None	

Please place an "X" next to the following statement to indicate your agreement:

_X_ I certify that I have answered every question and have not altered the wording of any of the questions on this form.

ICMJE DISCLOSURE FORM

Date:__June 6, 2021 ____
Your Name:__Yi-Ming Chen ____
Manuscript Title:__ ECP Overexpression in T Cells and Exosomes Induces IFN-γ Production and Tissue Inflammation
Manuscript number (if known):__ ar-20-2148 ____

In the interest of transparency, we ask you to disclose all relationships/activities/interests listed below that are related to the content of your manuscript. "Related" means any relation with for-profit or not-for-profit third parties whose interests may be affected by the content of the manuscript. Disclosure represents a commitment to transparency and does not necessarily indicate a bias. If you are in doubt about whether to list a relationship/activity/interest, it is preferable that you do so.

The following questions apply to the author's relationships/activities/interests as they relate to the current manuscript only.

The author's relationships/activities/interests should be defined broadly. For example, if your manuscript pertains to the epidemiology of hypertension, you should declare all relationships with manufacturers of antihypertensive medication, even if that medication is not mentioned in the manuscript.

In item #1 below, report all support for the work reported in this manuscript without time limit. For all other items, the time frame for disclosure is the past 36 months.

		**Name all entities with whom you have this relationship or indicate none (add rows as needed)**	**Specifications/Comments (e.g., if payments were made to you or to your institution)**
		**Time frame: Since the initial planning of the work**	
1	All support for the present manuscript (e.g., funding, provision of study materials, medical writing, article processing charges, etc.) **No time limit for this item.**	_X_ None	
		**Time frame: past 36 months**	
2	Grants or contracts from any entity (if not indicated in item #1 above).	_X__ None	
3	Royalties or licenses	__ X __ None	

4	Consulting fees	__ X __ None	
5	Payment or honoraria for lectures, presentations, speakers bureaus, manuscript writing or educational events	__ X __ None	
6	Payment for expert testimony	__ X __ None	
7	Support for attending meetings and/or travel	__ X __ None	
8	Patents planned, issued or pending	__ X __ None	
9	Participation on a Data Safety Monitoring Board or Advisory Board	__ X __ None	
10	Leadership or fiduciary role in other board, society, committee or advocacy group, paid or unpaid	__ X __ None	
11	Stock or stock options	__ X __ None	
12	Receipt of equipment, materials, drugs, medical writing, gifts or other services	___X _ None	
13	Other financial or non-financial interests	__ X __ None	

Please place an "X" next to the following statement to indicate your agreement:

_X_ I certify that I have answered every question and have not altered the wording of any of the questions on this form.

ICMJE DISCLOSURE FORM

Date:__June 6, 2021 ____
Your Name:__ Yi-Ru Ciou____
Manuscript Title:__ ECP Overexpression in T Cells and Exosomes Induces IFN-γ Production and Tissue Inflammation
Manuscript number (if known):__ ar-20-2148 ____

In the interest of transparency, we ask you to disclose all relationships/activities/interests listed below that are related to the content of your manuscript. "Related" means any relation with for-profit or not-for-profit third parties whose interests may be affected by the content of the manuscript. Disclosure represents a commitment to transparency and does not necessarily indicate a bias. If you are in doubt about whether to list a relationship/activity/interest, it is preferable that you do so.

The following questions apply to the author's relationships/activities/interests as they relate to the current manuscript only.

The author's relationships/activities/interests should be defined broadly. For example, if your manuscript pertains to the epidemiology of hypertension, you should declare all relationships with manufacturers of antihypertensive medication, even if that medication is not mentioned in the manuscript.

In item #1 below, report all support for the work reported in this manuscript without time limit. For all other items, the time frame for disclosure is the past 36 months.

		**Name all entities with whom you have this relationship or indicate none (add rows as needed)**	**Specifications/Comments (e.g., if payments were made to you or to your institution)**
		**Time frame: Since the initial planning of the work**	
1	All support for the present manuscript (e.g., funding, provision of study materials, medical writing, article processing charges, etc.) **No time limit for this item.**	_ X _ None	
		**Time frame: past 36 months**	
2	Grants or contracts from any entity (if not indicated in item #1 above).	_X__ None	
3	Royalties or licenses	__ X __ None	

4	Consulting fees	__ X __ None	
5	Payment or honoraria for lectures, presentations, speakers bureaus, manuscript writing or educational events	__ X __ None	
6	Payment for expert testimony	__ X __ None	
7	Support for attending meetings and/or travel	__ X __ None	
8	Patents planned, issued or pending	__ X __ None	
9	Participation on a Data Safety Monitoring Board or Advisory Board	__ X __ None	
10	Leadership or fiduciary role in other board, society, committee or advocacy group, paid or unpaid	__ X __ None	
11	Stock or stock options	__ X __ None	
12	Receipt of equipment, materials, drugs, medical writing, gifts or other services	___X _ None	
13	Other financial or non-financial interests	__ X __ None	

Please place an "X" next to the following statement to indicate your agreement:

_X_ I certify that I have answered every question and have not altered the wording of any of the questions on this form.

ICMJE DISCLOSURE FORM

Date:__June 6, 2021 ____
Your Name:__ Chia-Hsin Hsueh **____**
Manuscript Title:__ ECP Overexpression in T Cells and Exosomes Induces IFN-γ Production and Tissue Inflammation
Manuscript number (if known):__ ar-20-2148 ____

In the interest of transparency, we ask you to disclose all relationships/activities/interests listed below that are related to the content of your manuscript. "Related" means any relation with for-profit or not-for-profit third parties whose interests may be affected by the content of the manuscript. Disclosure represents a commitment to transparency and does not necessarily indicate a bias. If you are in doubt about whether to list a relationship/activity/interest, it is preferable that you do so.

The following questions apply to the author's relationships/activities/interests as they relate to the current manuscript only.

The author's relationships/activities/interests should be defined broadly. For example, if your manuscript pertains to the epidemiology of hypertension, you should declare all relationships with manufacturers of antihypertensive medication, even if that medication is not mentioned in the manuscript.

In item #1 below, report all support for the work reported in this manuscript without time limit. For all other items, the time frame for disclosure is the past 36 months.

		**Name all entities with whom you have this relationship or indicate none (add rows as needed)**	**Specifications/Comments (e.g., if payments were made to you or to your institution)**
**Time frame: Since the initial planning of the work**			
1	All support for the present manuscript (e.g., funding, provision of study materials, medical writing, article processing charges, etc.) **No time limit for this item.**	_ X _ None	
**Time frame: past 36 months**			
2	Grants or contracts from any entity (if not indicated in item #1 above).	_X__ None	
3	Royalties or licenses	__ X __ None	

4	Consulting fees	__ X __ None	
5	Payment or honoraria for lectures, presentations, speakers bureaus, manuscript writing or educational events	__ X __ None	
6	Payment for expert testimony	__ X __ None	
7	Support for attending meetings and/or travel	__ X __ None	
8	Patents planned, issued or pending	__ X __ None	
9	Participation on a Data Safety Monitoring Board or Advisory Board	__ X __ None	
10	Leadership or fiduciary role in other board, society, committee or advocacy group, paid or unpaid	__ X __ None	
11	Stock or stock options	__ X __ None	
12	Receipt of equipment, materials, drugs, medical writing, gifts or other services	___X _ None	
13	Other financial or non-financial interests	__ X __ None	

Please place an "X" next to the following statement to indicate your agreement:

_X_ I certify that I have answered every question and have not altered the wording of any of the questions on this form.

ICMJE DISCLOSURE FORM

Date:__June 6, 2021 ____
Your Name:__ Ching-Yi Tsai____
Manuscript Title:__ ECP Overexpression in T Cells and Exosomes Induces IFN-γ Production and Tissue Inflammation
Manuscript number (if known):__ ar-20-2148 ____

In the interest of transparency, we ask you to disclose all relationships/activities/interests listed below that are related to the content of your manuscript. "Related" means any relation with for-profit or not-for-profit third parties whose interests may be affected by the content of the manuscript. Disclosure represents a commitment to transparency and does not necessarily indicate a bias. If you are in doubt about whether to list a relationship/activity/interest, it is preferable that you do so.

The following questions apply to the author's relationships/activities/interests as they relate to the <u>current manuscript only</u>.

The author's relationships/activities/interests should be <u>defined broadly</u>. For example, if your manuscript pertains to the epidemiology of hypertension, you should declare all relationships with manufacturers of antihypertensive medication, even if that medication is not mentioned in the manuscript.

In item #1 below, report all support for the work reported in this manuscript without time limit. For all other items, the time frame for disclosure is the past 36 months.

		**Name all entities with whom you have this relationship or indicate none (add rows as needed)**	**Specifications/Comments (e.g., if payments were made to you or to your institution)**
		**Time frame: Since the initial planning of the work**	
1	All support for the present manuscript (e.g., funding, provision of study materials, medical writing, article processing charges, etc.)<br>**No time limit for this item.**	_ X _ None	
		**Time frame: past 36 months**	
2	Grants or contracts from any entity (if not indicated in item #1 above).	_X__ None	
3	Royalties or licenses	__ X __ None	

4	Consulting fees	__ X __ None	
5	Payment or honoraria for lectures, presentations, speakers bureaus, manuscript writing or educational events	__ X __ None	
6	Payment for expert testimony	__ X __ None	
7	Support for attending meetings and/or travel	__ X __ None	
8	Patents planned, issued or pending	__ X __ None	
9	Participation on a Data Safety Monitoring Board or Advisory Board	__ X __ None	
10	Leadership or fiduciary role in other board, society, committee or advocacy group, paid or unpaid	__ X __ None	
11	Stock or stock options	__ X __ None	
12	Receipt of equipment, materials, drugs, medical writing, gifts or other services	___X _ None	
13	Other financial or non-financial interests	__ X __ None	

Please place an "X" next to the following statement to indicate your agreement:

_X_ I certify that I have answered every question and have not altered the wording of any of the questions on this form.

ICMJE DISCLOSURE FORM

Date:__June 6, 2021 ____
Your Name:__Tse-Hua Tan ____
Manuscript Title:__ ECP Overexpression in T Cells and Exosomes Induces IFN-γ Production and Tissue Inflammation
Manuscript number (if known):__ ar-20-2148 ____

In the interest of transparency, we ask you to disclose all relationships/activities/interests listed below that are related to the content of your manuscript. "Related" means any relation with for-profit or not-for-profit third parties whose interests may be affected by the content of the manuscript. Disclosure represents a commitment to transparency and does not necessarily indicate a bias. If you are in doubt about whether to list a relationship/activity/interest, it is preferable that you do so.

The following questions apply to the author's relationships/activities/interests as they relate to the current manuscript only.

The author's relationships/activities/interests should be defined broadly. For example, if your manuscript pertains to the epidemiology of hypertension, you should declare all relationships with manufacturers of antihypertensive medication, even if that medication is not mentioned in the manuscript.

In item #1 below, report all support for the work reported in this manuscript without time limit. For all other items, the time frame for disclosure is the past 36 months.

		**Name all entities with whom you have this relationship or indicate none (add rows as needed)**	**Specifications/Comments (e.g., if payments were made to you or to your institution)**
		**Time frame: Since the initial planning of the work**	
1	All support for the present manuscript (e.g., funding, provision of study materials, medical writing, article processing charges, etc.) **No time limit for this item.**	__ None	
		Grants from National Health Research Institutes, Taiwan	IM-107-PP-01 and IM-107-SP-01
		Grants from Ministry of Science and Technology, Taiwan	MOST-106-2321-B-400-013
		**Time frame: past 36 months**	
2	Grants or contracts from any entity (if not indicated in item #1 above).	_X__ None	

3	Royalties or licenses	__ X __ None	
4	Consulting fees	__ X __ None	
5	Payment or honoraria for lectures, presentations, speakers bureaus, manuscript writing or educational events	__ X __ None	
6	Payment for expert testimony	__ X __ None	
7	Support for attending meetings and/or travel	__ X __ None	
8	Patents planned, issued or pending	__ X __ None	
9	Participation on a Data Safety Monitoring Board or Advisory Board	__ X __ None	
10	Leadership or fiduciary role in other board, society, committee or advocacy group, paid or unpaid	__ X __ None	
11	Stock or stock options	__ X __ None	
12	Receipt of equipment, materials, drugs, medical writing, gifts or other services	___X _ None	
13	Other financial or non-financial interests	__ X __ None	

Please place an "X" next to the following statement to indicate your agreement:

_X_ I certify that I have answered every question and have not altered the wording of any of the questions on this form.
